# Oncogenic RAS-induced CK1α drives nuclear FOXO proteolysis

**DOI:** 10.1038/onc.2017.334

**Published:** 2017-09-25

**Authors:** F Zhang, D M Virshup, J K Cheong

**Affiliations:** 1 Programme in Cancer and Stem Cell Biology, Duke-NUS Medical School, Singapore; 2 Department of Biochemistry, National University of Singapore, Singapore; 3 Department of Pediatrics, Duke University School of Medicine, Durham, NC, USA

## Abstract

Evasion of forkhead box O (FOXO) family of longevity-related transcription factors-mediated growth suppression is necessary to promote cancer development. Since somatic alterations or mutations and transcriptional dysregulation of the *FOXO* genes are infrequent in human cancers, it remains unclear how these tumour suppressors are eliminated from cancer cells. The protein stability of FOXO3A is regulated by Casein Kinase 1 alpha (CK1α) in an oncogenic RAS-specific manner, but whether this mode of regulation extends to related FOXO family members is unknown. Here we report that CK1α similarly destabilizes FOXO4 in RAS-mutant cells by phosphorylation at serines 265/268. The CK1α-dependent phosphoregulation of FOXO4 is primed, in part, by the PI3K/AKT effector axis of oncogenic RAS signalling. In addition, mutant RAS coordinately elevates proteasome subunit expression and proteolytic activity to eradicate nuclear FOXO4 proteins from RAS-mutant cancer cells. Importantly, dual inhibition of CK1α and the proteasome synergistically inhibited the growth of multiple RAS-mutant human cancer cell lines of diverse tissue origin by blockade of nuclear FOXO4 degradation and induction of caspase-dependent apoptosis. Our findings challenge the current paradigm that nuclear export regulates the proteolysis of FOXO3A/4 tumour suppressors in the context of cancer and illustrates how oncogenic RAS-mediated degradation of FOXOs, via post-translational mechanisms, blocks these important tumour suppressors.

## Introduction

The forkhead box O (FOXO) family of longevity-related transcription factors, in particular, FOXO1, FOXO3 and FOXO4, regulates a myriad of cellular processes that include nutrient metabolism,^1, 2, 3^ DNA damage response,^4^ oxidative stress response,^5^ autophagy,^1, 6, 7^ cell differentiation,^8, 9^ cell cycle progression^4, 10^ and cell death.^11, 12, 13, 14, 15^ Although cell culture-based molecular and biochemical studies suggest functional redundancy among the FOXO proteins, somatic deletion of the respective *FoxOs* in mice revealed unique physiological roles of the FoxOs *in vivo*. While FoxO1 is required for vasculogenesis,^16, 17^ FoxO3 plays a critical role in ovarian primordial follicle activation.^17, 18^ FoxO4-null mice exhibit increased intestinal epithelial permeability and are susceptible to trinitrobenzene sulfonic acid (TNBS)-induced colitis.^19^ In the context of cancer, it remains controversial whether FOXOs act as bona fide tumour suppressors. For instance, the respective *FoxO* knockout mice exhibit little or no incidence of spontaneous tumours.^17^ However, conditional compound deletion of *FoxO1*, *FoxO3* and *FoxO4* in mice resulted in the development of spontaneous lymphomas and hemangiomas, indicating that FOXOs are functionally redundant growth suppressors.^9^ *FOXO1* and *FOXO3* have also been recently identified to be targets of recurrent point mutations or homozygous deletions in a subset of human lymphoid neoplasms^20, 21^ and breast cancers,^22^ suggesting that evasion of FOXO-mediated growth suppression is necessary to promote cancer initiation/progression in a subset of tissue types. While mouse knockout studies suggest its importance as a tumour suppressor, whether FOXO4 is altered in a broad range of human cancers is currently unknown.

The activation of RAS signalling by extracellular growth factors or somatic mutation of RAS isoforms and/or its downstream effectors has been implicated in the control of subcellular localization or protein stability of multiple FOXO isoforms.^11, 12, 23, 24, 25, 26, 27^ Multiple kinases associated with the effector pathways of RAS signalling, such as the rapidly accelerated fibrosarcoma (RAF) kinase, phosphoinositide-3 kinase (PI3K), and Ral guanine nucleotide dissociation stimulator (RalGDS) signalling circuits, have also been shown to regulate the function of FOXO proteins via post-translational modifications. Upon the activation of insulin signalling, Protein Kinase B (PKB, commonly known as AKT) or the closely related serum and glucocorticoid-induced kinase (SGK) directly phosphorylate FOXO proteins at three evolutionarily conserved serine/threonine residues to induce nuclear export and thereby block the transcriptional activity of FOXOs.^11, 12, 23, 25^ Conversely, oxidative stress can promote Ral/JNK-mediated phosphorylation of FOXO4, resulting in increased nuclear translocation of FOXO4 and transactivation of FOXO4-responsive genes.^5, 24^ Furthermore, several studies have also identified RAS effector kinases that directly control the transcriptional activity or turnover of FOXO proteins.^27, 28, 29, 30^

Although multiple mechanisms exist to regulate the activity of FOXO family members, their relative importance in cancer is not well understood. We recently demonstrated that mutant RAS, via its PI3K/AKT/mTOR effector signalling axis, upregulates the protein abundance of a ubiquitously expressed serine/threonine kinase, Casein Kinase 1 alpha (CK1α).^29^ We further showed that CK1α, but not CK1δ or CK1ε, phosphorylates and destabilizes nuclear FOXO3A to tightly regulate the level of basal autophagy in RAS-mutant cancer cells. Our data are consistent with earlier studies that reported CK1-mediated phosphorylation of FOXO1 *in vitro*^31, 32^ and it prompted us to investigate whether CK1α and/or other CK1 isoforms also phosphorylate the less well-characterized FOXO4 to modulate its function(s) *in vivo*. Since the CK1α phosphorylation motif that we identified in FOXO3A is conserved in FOXO1 and FOXO4,^29^ we hypothesized that CK1α-dependent phosphoregulation of FOXO4 targets it for increased protein turnover to promote the growth/survival of RAS-mutant cancer cells. Using mutant K-RAS isogenic human colon cancer cell lines, we find that the abundance of CK1α and FOXO proteins is inversely correlated in an oncogenic RAS-specific manner. We further demonstrate that CK1α-dependent phosphorylation of FOXO4 at serine residues 265 and 268 is necessary for 26 S proteasome-mediated FOXO4 proteolysis in the nuclei of K-RAS-mutant colon cancer cells. Simultaneous gain in proteasome function appears to be important as well, as we observed upregulation of Nrf1, Nrf2 and proteasomal subunit expression as well as elevated proteasome activity in a mutant RAS-specific manner. Notably, dual inhibition of CK1α and the proteasome synergistically inhibited the growth of RAS-mutant cancer cells of diverse tissue origin. Demonstrating the importance of this pathway, forced expression of CK1α phospho-acceptor site mutant of FOXO4 (FOXO4^S265/268A^) potently halted the growth of RAS-mutant colon cancer cells by inducing apoptosis. Our data are consistent with the recently reported tumour suppressive role of FOXO4 in human gastrointestinal (GI) cancers^33, 34, 35^ and supports a strategy of targeting CK1α via pharmacological means to combat a subset of cancers, particularly those with activating mutations of RAS and/or activation of its diverse effector signalling cascades.

## Results

### FOXO isoforms are downregulated in an oncogenic RAS-specific manner

**Figure 1:**
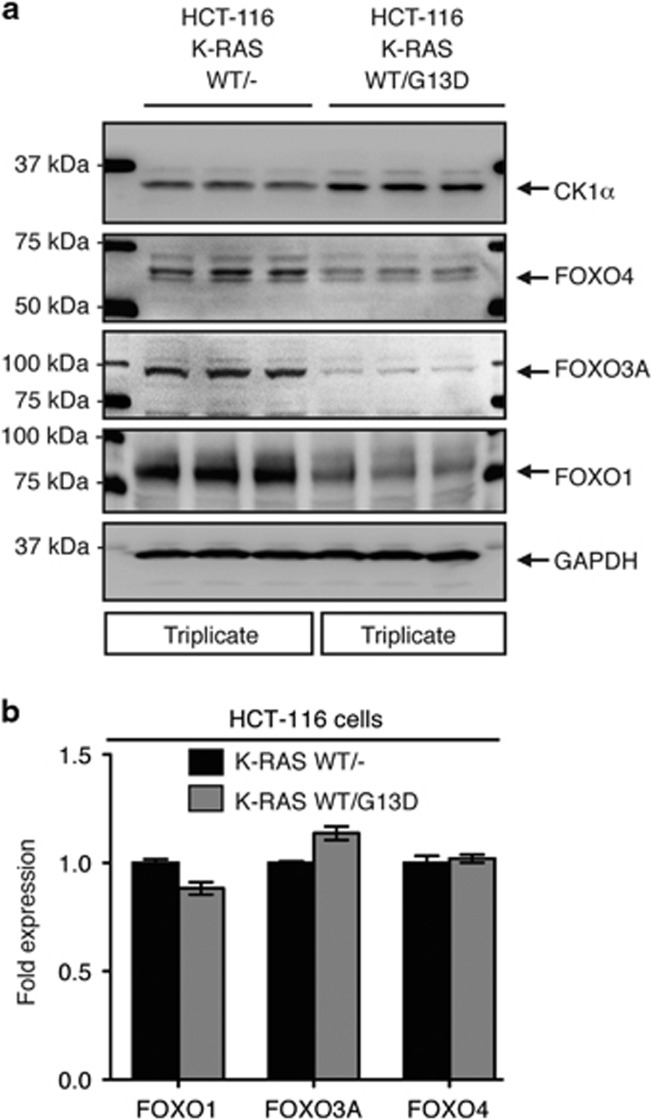
Protein, not mRNA, abundance of FOXO isoforms is downregulated specifically in RAS-mutant colon cancer cells. (**a**) Representative immunoblots of endogenous CK1α, FOXO1, FOXO3A and FOXO4 protein expression in the HCT-116 K-RAS isogenic cell lines. GAPDH serves as the loading control. (**b**) RT-qPCR profiling of *FOXO1*, *FOXO3A* and *FOXO4* mRNA expression in the HCT-116 K-RAS isogenic cell lines. Expression change in the *FOXO* transcripts was first normalized with *PGK* and *HPRT* expression in the respective cell line. Fold expression change in normalized *FOXO* expression in HCT-116 K-RAS (WT/G13D) cells was then calculated relative to normalized *FOXO* expression in HCT-116 K-RAS (WT/−) cells. Similar results were observed in three independent experiments.

Somatic alterations (or mutations) and transcriptional dysregulation of *FOXO* isoforms are infrequent in multiple human cancers, unlike other tumour suppressors such as TP53 (commonly known as p53) and Adenomatous polyposis coli (APC; Supplementary Figures 1a–d). We recently reported that oncogenic RAS (K-RAS^G13D^ and H-RAS^G12V^), via its PI3K/AKT/mTOR/CK1α effector pathway, downregulates FOXO3A protein abundance in human cancer cells. This is consistent with earlier reports that implicated aberrant RAS signalling in the control of subcellular localization or protein stability of multiple FOXO isoforms.^11, 12, 23, 24, 25, 26, 27^ Using the isogenic human colon cancer cells HCT-116 ^K-RAS WT/G13D^ and HCT-116 ^K-RAS WT/−^, where the oncogenic *K-RAS*^*G13D*^ allele has been knocked out by homologous recombination,^36^ we found that the protein but not mRNA abundance of other FOXO isoforms like FOXO1 and FOXO4 are also downregulated specifically in RAS-mutant human colon cancer cells (Figures 1a and b). Our findings suggest that RAS-mutant cancer cells reduce the activity of these growth suppressors by controlling their protein turnover.

### Protein abundance of FOXO4 is regulated by CK1α in a mutant RAS-specific manner

**Figure 2:**
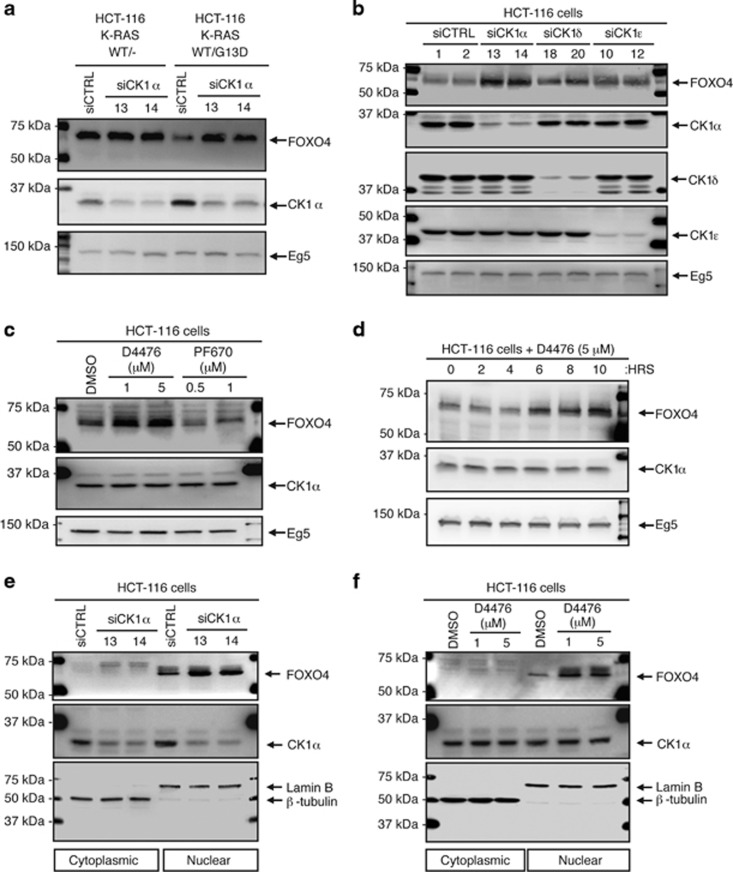
CK1α, not CK1δ or CK1ε, regulates FOXO4 protein abundance in a mutant RAS-specific manner. (**a**) CK1α depletion increases FOXO4 protein abundance in a mutant K-RAS-dependent manner. HCT-116 K-RAS isogenic cells were transfected with 100 nM siCtrl or siCK1α (#13 or #14) for 48 h prior to SDS–PAGE/western blot (WB) analysis using the indicated antibodies (*n*=3). (**b**) CK1α is the only non-membrane bound CK1 isoform that phosphorylates FOXO4 *in vivo*. Each of the indicated CK1 isoforms in HCT-116 cells was depleted with two independent siRNAs (48 h) prior to SDS–PAGE/WB analysis using the indicated antibodies (*n*=3). (**c**) Inhibition of CK1α, not CK1δ/ε, increases FOXO4 protein abundance. Cells were treated with the indicated compounds for 16 h prior to SDS–PAGE/WB analysis using the indicated antibodies (*n*=3). (**d**) CK1α inhibition increases FOXO4 protein abundance in a time-dependent manner. Cells were incubated with the indicated compound over 10 h prior to SDS–PAGE/WB analysis using the indicated antibodies (*n*=3). Eg5 serve as the loading control for **a**–**d**. (**e**) Depletion of CK1α by RNAi or (**f**) inhibition of CK1α by D4476 increases nuclear FOXO4 protein abundance. Cells were (**e**) transfected with the indicated siRNAs (100 nM; 48 h treatment) or (**f**) treated with the indicated drugs for 4 h prior to subcellular fractionation and SDS–PAGE/WB analysis using the indicated antibodies (*n*=3). Lamin B and β-tubulin serve as the loading control for nuclear and cytosolic fractions, respectively.

Given that CK1 has been shown to phosphorylate FOXO1 *in vitro* and FOXO3A *in vivo*,^29, 32^ we investigated whether CK1α or other CK1 isoforms regulate the protein abundance of the less well-characterized FOXO4 isoform. CK1α was depleted in the HCT-116 K-RAS isogenic cells via two independent siRNAs (siCK1α) and FOXO4 protein abundance was assessed. There was a mutant RAS-dependent increase of FOXO4 protein abundance in the CK1α-knockdown cells (Figure 2a). To determine whether this effect was isoform-specific, we depleted CK1α, CK1δ or CK1ε in HCT-116 cells via two independent siRNAs that target each of these CK1 isoforms. siRNA depletion of CK1α, but not CK1δ or CK1ε, led to a dramatic increase in FOXO4 protein abundance (Figure 2b). Furthermore, pharmacological inhibition of CK1α/δ/ε by D4476 (Supplementary figure 2a),^32, 37, 38, 39, 40^ but not PF670462 (PF670)-mediated specific inhibition of CK1δ/ε,^41^ increased FOXO4 protein abundance in HCT-116 cells (Figure 2c), suggesting that CK1α kinase activity is required for FOXO4 protein turnover. Notably, we show that D4476 is a potent inhibitor of CK1α1/δ/ε and is significantly less active against CK1α1L and CK1γ1–3 (Supplementary figure 2a). The measured IC_50_ values of D4476 against CK1α, CK1δ and CK1ε in the presence of 10 μM ATP are 225 nM, 39.25 nM and 159.6 nM respectively. Similarly, FOXO4 protein abundance increased upon siRNA depletion of CK1α or pharmacological inhibition of CK1α by D4476 in another RAS-mutant colon cancer cell line, SW480 (Supplementary Figure 2b). FOXO4 proteins accumulate in D4476-treated HCT-116 cells in a time-dependent manner (Figure 2d).

To study whether the cytoplasmic or nuclear pool of endogenous FOXO4 proteins was altered by CK1α loss-of-function, subcellular fractionation of lysates from HCT-116 cells that were treated with siCK1α or D4476 was performed (Figures 2e and f). The faster migrating but more abundant FOXO4 proteins were localized to the nucleus, and the less predominant slower migrating FOXO4 proteins were localized to the cytoplasm. Notably, siRNA depletion or pharmacological inhibition of CK1α further increased the faster migrating FOXO4 proteins in the nucleus of HCT-116 cells (Figures 2e and f). Consistent with the mobility shift being due to phosphorylation, the slower migrating species of FOXO4 were completely abolished when HCT-116 and SW480 cell lysates were treated with alkaline phosphatase *in vitro* (Supplementary figure 2c). Taken together, the data indicate that endogenous FOXO4 proteins are predominantly localized to the nucleus of RAS-mutant HCT-116 cells and are destabilized, rather than exported, dependent on nuclear CK1α activity. This suggests a dominant role of protein degradation, rather than nuclear export, in the regulation of FOXO4 proteostasis in these RAS-mutant cancer cells.

### CK1α phosphorylates FOXO4 proteins specifically at serine residues 265 and 268 *in vivo*

**Figure 3:**
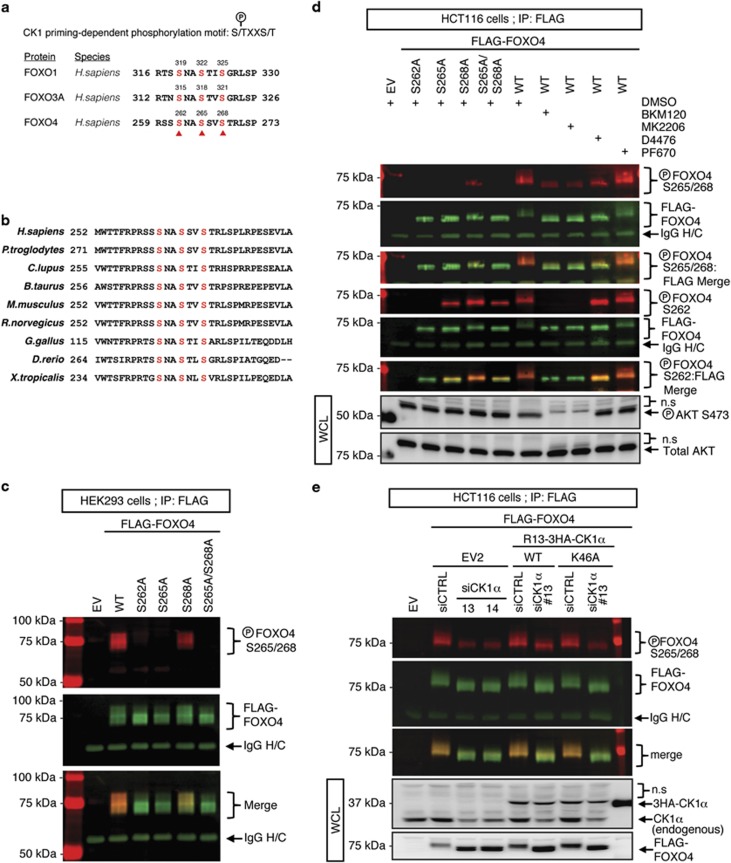
CK1α phosphorylates FOXO4 proteins specifically at serine residues 265 and 268 *in vivo*. (**a**) CLUSTALW protein sequence alignment of the FOXO isoforms revealed that the conserved CK1 phosphorylation motif is present in FOXO4. (**b**) NCBI HomoloGene protein sequence alignment of FOXO4 across different species indicates that the CK1α phosphorylation motif of FOXO4 is evolutionarily conserved. (**c**) FOXO4 is phosphorylated at S265 and S268 *in vivo*. Cells transiently expressing the indicated proteins for 48 h were assessed by IP and WB. FOXO4 IP was analyzed by SDS–PAGE /WB using the red channel of LI-COR for phosphoepitope antibodies and the green channel for FLAG-FOXO4. (**d**) Inhibition of PI3K, AKT, or CK1α markedly reduces FOXO4^S265/268^ phosphorylation. Cells transiently expressing the indicated proteins for 48 h were treated with BKM120 (5 μM), MK2206 (5 μM), D4476 (5 μM) or PF670 (1 μM) for 4 h. FOXO4 IP was analyzed by SDS–PAGE /WB (LI-COR and enhanced chemiluminescence) with the indicated antibodies. (**e**) Depletion of CK1α markedly reduces FOXO4^S265/268^ phosphorylation. Cells were transiently transfected with the indicated plasmids for 24 h. They were then transfected with the indicated siRNAs for an additional 48 h, followed by IP and SDS–PAGE /WB (LI-COR and enhanced chemiluminescence) analysis using the indicated antibodies. For **c**–**e**, merged LI-COR immunoblot panels represent the combined fluorescence signals from paired sets of p-FOXO4^S262 or S265/268^ and FLAG-FOXO4. IgG H/C: IgG heavy chain; WCL: Whole cell lysate; n.s: non-specific bands. Similar results were observed in at least two independent experiments with duplicates.

To gain a mechanistic understanding on how CK1α regulates FOXO4 protein turnover, we performed CLUSTALW multiple protein sequence alignment of the FOXO isoforms and found that the CK1 phosphorylation motif pS/pT–X–X–S/T (where pS/pT refers to a phospho-serine or phospho-threonine, and X refers to any amino acid) is also present in FOXO4 (Figure 3a). This CK1 phosphorylation motif is evolutionarily conserved across different species (Figure 3b), suggesting that CK1-dependent phosphoregulation of FOXO4 might be important for its function(s). Based on the identified CK1 phosphorylation motif, we postulated that the serine 262 residue (S262) of FOXO4 could be phosphorylated by a priming kinase allowing subsequent CK1α-mediated phosphorylation of the serine 265 and 268 residues (S265, S268) of FOXO4. To investigate whether CK1α directly phosphorylates FOXO4 proteins in RAS-mutant cancer cells, we generated a phospho-specific antibody that targets phosphorylated S265 and S268 of FOXO4 (p-FOXO4^S265/268^) (Figure 3c). Single and double alanine mutants of S262, S265 and S268 of FLAG-FOXO4 (S262A, S265A, S268A and S265/268A) were generated to validate the specificity of this antibody. As Figure 3c shows, the p-FOXO4^S265/268^ antibody reacted specifically with the FLAG-FOXO4^WT^ and partially with FLAG-FOXO4^S268A^, but not with FLAG-FOXO4^S265A^ or FLAG-FOXO4^S265/268A^ proteins. The data suggest that both S265 and S268 of FOXO4 are phosphorylated *in vivo*. Notably, we also observed that FOXO4^S265/268^ phosphorylation was completely abolished in the putative priming site mutant, S262A (Figure 3c), consistent with the model that FOXO4^S265/268^ phosphorylation is mainly dependent on the priming phosphorylation at FOXO4^S262^.

Given that this priming serine residue is part of a conserved AKT phosphorylation motif (R-X-R-X-X-pS/pT) among the FOXO isoforms (Figure 3a) and its phosphorylation by AKT is required for CK1-mediated phosphorylation of FOXO1 and FOXO3A proteins,^26, 29^ we investigated whether AKT phosphorylates FOXO4 at S262 to prime subsequent CK1α-mediated FOXO4^S265/268^ phosphorylation (Supplementary Figure 3a). Notably, pharmacologic inhibition of AKT by MK2206 or its upstream activator PI3K by BKM120 markedly reduced S262 phosphorylation of FLAG-FOXO4^WT^ (Supplementary Figure 3a), confirming that AKT serves as the priming kinase to promote CK1α-dependent phosphorylation of FOXO4 *in vivo*. Furthermore, inhibition of PI3K or AKT in the RAS-mutant HCT-116 cells abolished the phosphorylation-associated gel mobility shift of endogenous FOXO4 proteins (Supplementary figure 3b). Importantly, pharmacological inhibition of PI3K, AKT, CK1α, but not CK1δ/ε, markedly reduced FOXO4^S265/268^ phosphorylation (Figure 3d). While we confirmed that blockade of AKT (by MK2206) or its upstream activator PI3K (by BKM120) abolished S262 phosphorylation of FLAG-FOXO4^WT^, inhibition of CK1α (by D4476) did not result in loss of S262 phosphorylation of FLAG-FOXO4^WT^ (Figure 3d). Via immunoprecipitation (IP) *in vitro* kinase assays, we further demonstrated that recombinant human AKT1 and CK1α1 directly phosphorylate S262 and S265/268 of alkaline phosphatase pretreated-FLAG-FOXO4^WT^ respectively (Supplementary Figure 3c–e). The absence of S265/268 phosphorylation of the phospho-acceptor mutant FOXO4^S262A^ following sequential incubation with recombinant human AKT1 and CK1α1 *in vitro* suggests that the priming phosphorylation of FOXO4^S262^ by AKT is required for CK1α-mediated phosphorylation of FOXO4^S265/268^ (Supplementary Figure 3d). Notably, we showed that CK1α-dependent FOXO4^S265/268^ phosphorylation occurs only after prior AKT-mediated FOXO4^S262^ phosphorylation (Supplementary Figure 3e). Depletion of CK1α by two independent CK1α-specific siRNAs (siCK1α) also markedly reduced FOXO4^S265/268^ phosphorylation and this was partially rescued by re-expression of siRNA-resistant (R13) wildtype (WT) but not kinase-dead (K46A) CK1α (Figure 3e). We further confirmed that phospho-acceptor site mutagenesis as well as inhibition of AKT (by MK2206) or CK1α (by D4476) did not alter nuclear localization of FLAG-FOXO4 (Supplementary Figure 3f). Similar results were observed for phospho-acceptor mutants of HA-FOXO3A (Supplementary Figure 3f). Collectively, our data indicate that CK1α specifically phosphorylates FOXO4 at S265 and S268, and this requires the priming phosphorylation at S262 by AKT.

### CK1α-dependent phosphorylation of FOXO4 is necessary for 26 S proteasome-mediated FOXO4 proteolysis in the nuclei of RAS-mutant colon cancer cells

**Figure 4:**
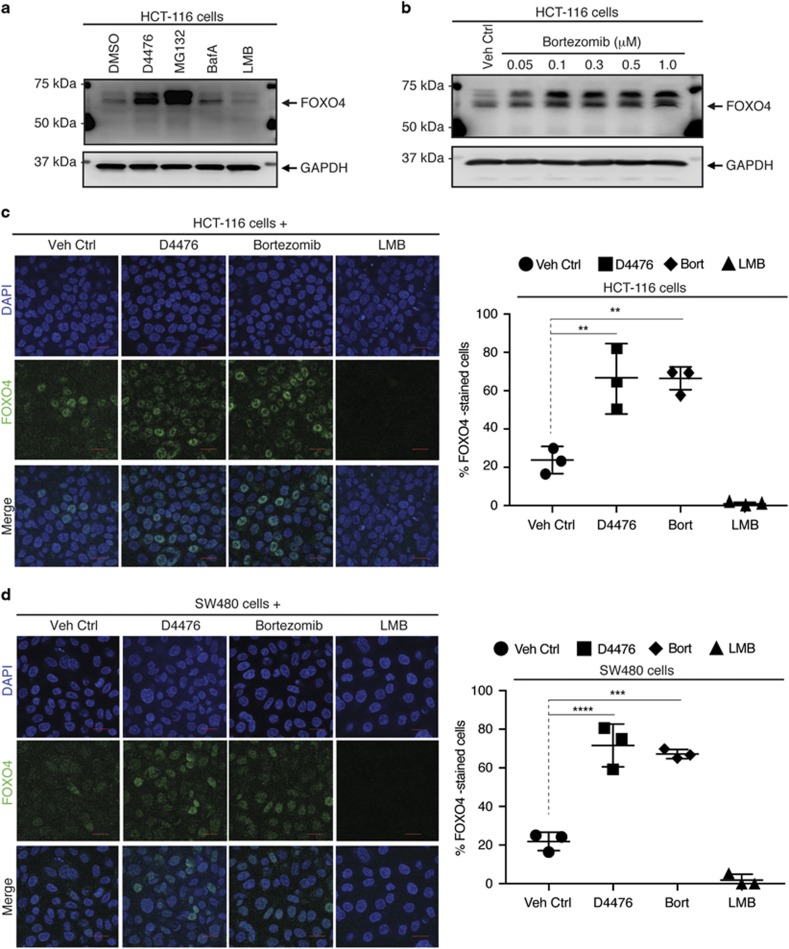
Pharmacological inhibition of CK1α or 26 S proteasome increases nuclear FOXO4 protein abundance. (**a**) Opposing effects of D4476 or MG132 and LMB on FOXO4 protein abundance. Cells were treated with DMSO, D4476 (5 μM), MG132 (20 μM), Baf A (1 μM) or LMB (20 nM) for 4 h prior to cell lysis for SDS–PAGE /WB analysis (*n*=3). (**b**) Bortezomib elevates FOXO4 protein abundance in a dose-dependent manner. Cells were treated with DMSO or the indicated doses of Bortezomib for 4 h prior to cell lysis for SDS–PAGE /WB analysis with the indicated antibodies (*n*=3). (**c**, **d**) Inhibition of CK1α or 26 S proteasome upregulates nuclear FOXO4 protein abundance. (**c**) HCT-116 or (**d**) SW480 cells were treated with DMSO, D4476 (5 μM), Bortezomib (100 nM; Bort) or LMB (20 nM) for 4 h prior to immunofluorescence staining using the FOXO4 antibody. DAPI stains the nuclei of cells. Scale bar: 10 μm. FOXO4-DAPI positivity (as a readout for nuclear FOXO4) in each treatment group was quantified by automatic particle counting function of the FIJI Image J software and the data were plotted using the GraphPad Prism software (mean±s.d.). Similar results were observed in three independent experiments. One-way ANOVA with Dunnett’s test was used to analyze statistical significance; **P<0.01; ***P<0.001; ****P<0.0001.

The intracellular degradation of a subset of proteins is a functional consequence of protein post-translational modifications, including phosphorylation and ubiquitylation.^42, 43^ The 26 S proteasome- and the lysosome-dependent proteolytic pathways are known to govern intracellular protein turnover.^42^ To determine whether either one or both of these pathways are involved in the regulation of FOXO3A and FOXO4 protein turnover in RAS-mutant cancer cells, we incubated HCT-116 and SW480 cells with the proteasome inhibitor MG132 or the lysosome/autolysosome inhibitor Bafilomycin A (Baf A). While Baf A only modestly increased FOXO4 protein abundance, MG132-treated cells had markedly increased FOXO4 protein abundance, similar to D4476-treated cells (Figure 4a; Supplementary Figure 4a). Similar results were observed for FOXO3A protein abundance (Supplementary Figure 4a and b). Consistent with a key role for the proteasome, the FDA-approved proteasome inhibitor Bortezomib also increased the abundance of both FOXO4 (Figure 4b) and FOXO3A (Supplementary Figure 4c) in a dose-dependent manner.

Nucleo-cytoplasmic shuttling of phosphorylated FOXO proteins has been reported to be important for their proteolytic degradation. However, blocking nuclear export with Leptomycin B (LMB) did not increase FOXO4 abundance, and in fact led to a decrease consistent with nuclear degradation (Figure 4a; Supplementary Figure 4a). Similar results were observed for FOXO3A protein abundance (Supplementary Figure 4b). To determine whether the cytoplasmic or nuclear abundance of FOXO4 proteins was altered, we performed subcellular fractionation and immunofluorescence staining of endogenous FOXO4 proteins in HCT-116 and SW480 cells that were treated with CK1α, 26 S proteasome or nuclear export inhibitor. Consistent with our subcellular fractionation data (Supplementary Figure 4d), we found endogenous FOXO4 proteins to be predominantly localized to the nucleus in ~20% of HCT-116 and SW480 cells (Figures 4c and d). When these cells were exposed to either D4476 or Bortezomib, ~70% of nuclei were stained positive for FOXO4 proteins (Figures 4c and d). However, endogenous FOXO4 proteins appeared to be completely eradicated from the LMB-treated cells (Figures 4c and d). Taken together, our data indicate that the FOXO protein turnover in these RAS-mutant colon cancer cells is predominantly regulated by the nuclear 26 S proteasome.

### LMB-induced degradation of nuclear FOXO4 proteins is reversed by CK1α or 26 S proteasome inhibition

**Figure 5:**
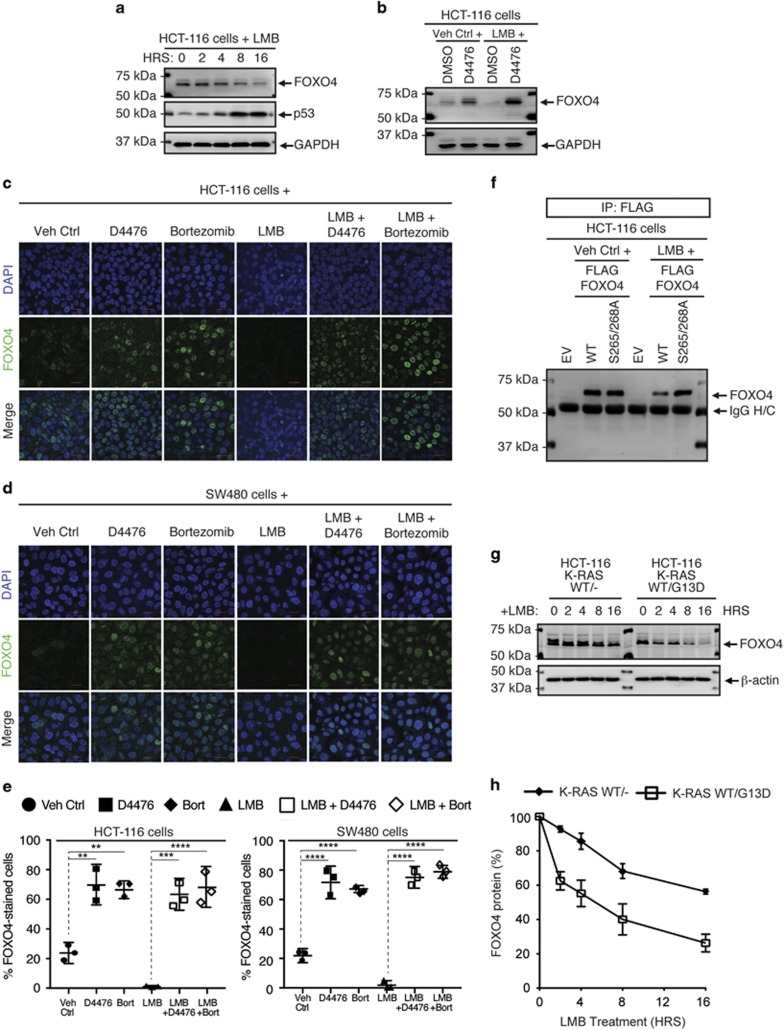
LMB-induced degradation of nuclear FOXO4 proteins is reversed by CK1α or 26 S proteasome inhibition. (**a**) LMB-blockade of nuclear export reduces FOXO4 protein abundance in a time-dependent manner. Cells were incubated with LMB (20 nM) over the indicated time, followed by cell lysis for SDS–PAGE /WB analysis using the indicated antibodies (*n*=3). HRS: hours. (**b**) CK1α inhibition abolishes LMB-induced FOXO4 proteolysis. Cells were incubated with DMSO or D4476 (5 μM), in the presence of vehicle control (Veh Ctrl; ethanol) or LMB (20 nM), for 4 h. Cells were lysed for SDS–PAGE /WB analysis using the indicated antibodies (*n*=3). (**c**, **d**) LMB-induced FOXO4 proteolysis is blocked by CK1α or 26 S proteasome inhibition. (**c**) HCT-116 or (**d**) SW480 cells were treated with Veh Ctrl (DMSO) or D4476 (5 μM), in the presence or absence of LMB (20 nM), for 4 h prior to immunofluorescence staining using the FOXO4 antibody. DAPI stains the nuclei of cells. Scale bar: 10 μm. FOXO4-DAPI positivity (as a readout for nuclear FOXO4) in each treatment group was quantified by automatic particle counting function of the FIJI Image J software. (**e**) Quantitation of immunofluorescence from drug-treated cells. Data obtained for the LMB+D4476 and LMB+Bortezomib (Bort) cohorts in the same experiments were added to the scatter plots that were originally presented in Figures 4c and d using GraphPad Prism (mean±s.d.). One-way ANOVA with Dunnett’s test was used to analyze statistical significance; **P<0.01; ***P<0.001; ****P<0.0001. (**f**) FLAG-FOXO4^S265/268A^ is resistant to LMB-induced proteolysis. Cells transiently expressing the indicated proteins for 48 h were treated with vehicle control (Veh Ctrl; ethanol) or LMB (20 nM) for 4 h. FOXO4 IP was analyzed by SDS–PAGE /WB with the indicated antibodies (*n*=3). (**g**) Enhanced proteolytic degradation rate of endogenous FOXO4 is mutant RAS-specific. HCT-116 K-RAS isogenic cell lines were incubated with DMSO or LMB (20 nM) over the indicated time followed by cell lysis for SDS–PAGE /WB analysis using the indicated antibodies (*n*=3). (**h**) Expression of FOXO4 is normalized with β-actin expression of each time point. Normalized FOXO4 expression in the LMB-treated samples of each cell line is plotted relative to that of its DMSO-treated samples using the GraphPad Prism software (mean±s.d.). Similar results were observed in three independent experiments.

Since FOXO4 proteins are markedly reduced when nuclear export is inhibited by LMB, we assessed the kinetics of LMB-induced FOXO4 proteolysis. FOXO4 protein abundance reduced after addition of LMB, with half-maximal decrease in 4 h (Figure 5a; Supplementary Figure 5a). Remarkably, the closely related FOXO3A proteins reduced 50% in 2 h in these RAS-mutant cells upon LMB treatment (Supplementary Figure 5b). We also showed that LMB induced the degradation of nuclear FOXO3A and FOXO4 proteins in a time-dependent manner (Supplementary Figure 5c). Confirming that the decrease of FOXO4 was not due to general toxicity of treatment, we noted that LMB increased the abundance of p53 proteins in the nuclei of these cells over time (Figure 5a; Supplementary Figure 5a and d), as previously reported.^44^

The data suggest that nuclear accumulation and phosphorylation of FOXO4 drives its degradation. Consistent with this, inactivation of CK1α by D4476 blocked the proteolysis of FOXO4 in LMB-treated cells (Figure 5b). Immunofluorescence staining of endogenous FOXO4 in these drug-treated cells further demonstrated that inhibition of CK1α (by D4476) or proteasome (by Bortezomib) is sufficient to block LMB-induced nuclear FOXO4 proteolysis (Figures 5c and e). This also fits the observation that LMB induced the proteolysis of FLAG-FOXO4^WT^, but not the CK1α-resistant FLAG-FOXO4^S265/268A^ (Figure 5f). To study the turnover of these FOXO4 variants more precisely, we blocked *de novo* protein synthesis of FLAG-FOXO4^WT^ or FLAG-FOXO4^S265/268A^—transfected HCT-116 cells using cycloheximide (CHX). We showed that FLAG-FOXO4^S265/268A^ proteins are indeed more resistant to 26 S proteasome-mediated degradation than their wildtype counterparts (Supplementary Figure 5e). Importantly, we demonstrated that the half-lives of endogenous FOXO4 as well as FOXO3A are relatively longer in LMB-treated HCT-116 K-RAS (WT/−) cells (Figures 5g and h; Supplementary Figure 5f–g), suggesting that mutant K-RAS is required for the destabilization of multiple FOXO tumour suppressors.

### Mutant RAS upregulates proteasomal subunit expression and proteasome activity

**Figure 6:**
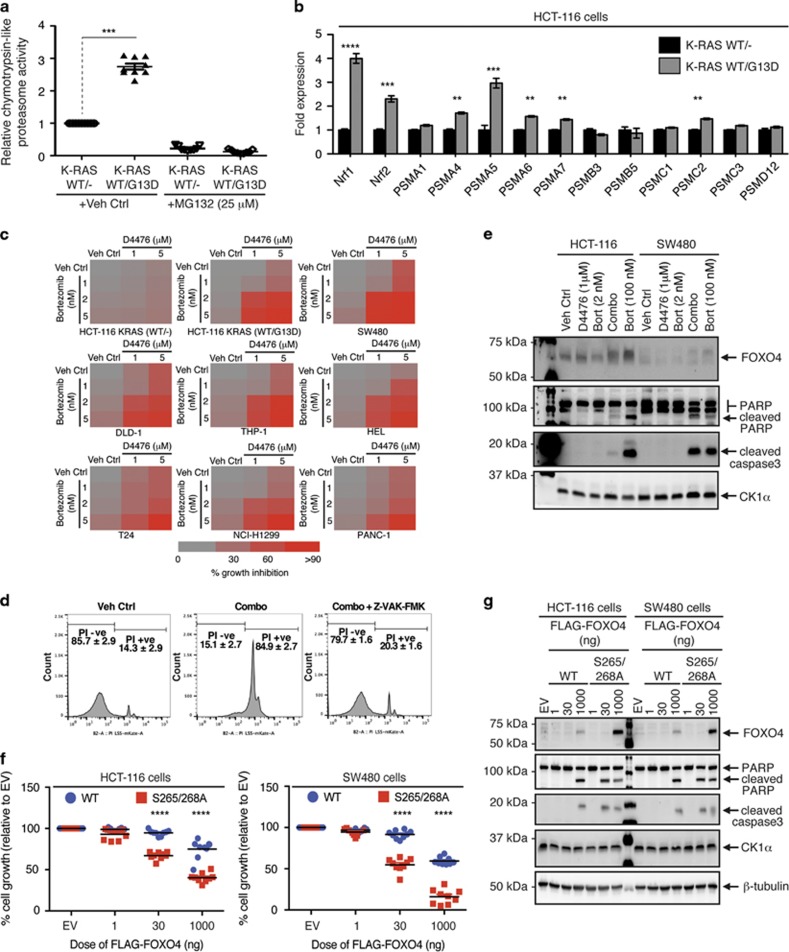
Dual inhibition of CK1α and proteasome blocked RAS-mutant cancer cell growth by activating caspase-dependent apoptosis. (**a**) Proteasome activity is enhanced specifically in RAS-mutant cancer cells. HCT-116 K-RAS isogenic cell lines were treated with vehicle control (Veh Ctrl; DMSO) or MG132 for 2 h, followed by chymotrypsin-like proteasome activity assays (mean±s.d.). (**b**) Expression of the transcription factors *Nrf1*, *Nrf2* as well as the *PSMA* and *PSMC* families of proteasome subunits are upregulated in a mutant RAS-specific manner. Gene expression was measured by RT-qPCR and shown as mean±s.d. (**c**) Dual CK1α and proteasome inhibition blocked the growth of RAS-mutant cancer cells of diverse tissue origin. The indicated cell lines were treated as indicated for 72 h. Percentage of cancer cell growth inhibition was assessed by crystal violet assays and is represented by a gain of red intensity in the two-color heat maps. (**d**) Dual CK1α and proteasome inhibition induced caspase-dependent apoptosis. HCT-116 cells were treated with Veh Ctrl, Combo (1 μM D4476+2 nM Bortezomib) or Combo with Z-VAD-FMK (100 μM) for 72 h, followed by flow cytometric quantitation of PI-stained cells (*n*=30 000 cells per treatment group in triplicates; mean±s.d.). (**e**) Accumulation of FOXO4 proteins correlate with cleavage of caspase 3 and PARP in drug combo-treated RAS-mutant colon cancer cells. Cells were incubated with the indicated drugs for 8 h prior to SDS–PAGE /WB analysis (*n*=3). Note: 100 nM Bortezomib treatment served as the positive control for apoptosis induction. (**f**) CK1α-resistant FOXO4 more actively inhibits cancer cell growth. Growth of WT- or S265/268 A-transfected cells (relative to that of EV-transfected cells) was assessed by crystal violet assays 5 days after transfection (*n*=9; data from three independent experiments with triplicate). (**g**) Ectopic expression of the CK1α-insensitive FOXO4 mutant induced apoptosis. Cells were transfected with the indicated amount of EV, WT or FOXO4^S265/268A^ plasmids 72 h prior to SDS–PAGE /WB analysis (*n*=3). For (**a**, **b** and **f**), one-way ANOVA with Bonferroni’s multiple comparisons test was used to analyze statistical significance; **P<0.01; ***P<0.001; ****P<0.0001; NS, not significant.

Given that the proteasome was identified as a common target in multiple oncogenic RAS synthetic lethal genetic screens,^45, 46, 47^ we hypothesized that RAS-mutant cancer cells also enhance proteasome activity to maintain proper proteostasis and to eradicate growth suppressors like FOXO3A and FOXO4. We measured the chymotrypsin-like activity of proteasome in the HCT-116 K-RAS isogenic cell lines via an *in vitro* fluorometric assay and found that the HCT-116 K-RAS (WT/G13D) cells possess approximately threefold higher proteasome activity than the HCT-116 K-RAS (WT/−) cells (Figure 6a). Notably, we observed that the gain of proteasome activity in HCT-116 K-RAS (WT/G13D) cells is confined to the nuclear as opposed to the cytoplasmic fraction (Supplementary Figure 6a). Since mutant RAS or its downstream effectors have been shown to upregulate Nrf1- and Nrf2-driven proteasome subunit expression,^48, 49^ we next determined whether the elevated proteasome activity in HCT-116 K-RAS (WT/G13D) cells is a direct consequence of elevated *Nrf1*, *Nrf2* and proteasome subunit expression. RT-qPCR profiling of gene expression of the HCT-116 K-RAS isogenic cell lines revealed an increase in expression of multiple core proteasome subunits, particularly *PSMA* and *PSMC* family members, in the HCT-116 K-RAS (WT/G13D) cells (Figure 6b). Notably, these RAS-mutant cells also expressed approximately four- and twofold more *Nrf1* and *Nrf2* transcripts, respectively (Figure 6b), suggesting that mutant RAS enhances the abundance of proteasomes by upregulating these cell stress-responsive transcription factors. Notably, the HCT-116 K-RAS (WT/G13D) cells showed greater sensitivity to low nanomolar doses of Bortezomib than the HCT-116 K-RAS (WT/−) cells (Supplementary Figure 6b), consistent with an earlier study that reported the differential killing of RAS-mutant cells by proteasome inhibitors.^50^ Our data indicate that the survival of RAS-mutant cells is critically dependent on enhanced proteasome expression and activity.

### Dual inhibition of CK1α and proteasome synergistically inhibited the growth of RAS-mutant cancer cells by inducing FOXO4 protein accumulation and caspase-dependent apoptosis

Since RAS-mutant cancer cells exhibit higher activity of both CK1α and the 20 S/26 S proteasome, we postulated that D4476 might potentiate the therapeutic efficacy of the clinically approved proteasome inhibitor Bortezomib. We treated a panel of nine RAS-wildtype and RAS-mutant cancer cells of diverse tissue origin with varying low micromolar doses of D4476 (1 and 5 μM), low nanomolar doses of Bortezomib (1, 2 and 5 nM) or their respective combinations and measured cell growth. We reasoned that the combination of sub-optimal doses of D4476 and Bortezomib could reduce undesirable off-target effects and prevent the development of adaptive resistance to these drugs. We observed that a subset of the D4476:Bortezomib combinations synergistically inhibited the growth of HCT-116 K-RAS (WT/G13D) and other RAS-mutant cancer cells (SW480, DLD-1, THP-1, HEL, T24, NCI-H1299 and PANC-1) but not HCT-116 K-RAS (WT/−) cells (Figure 6c; Supplementary Figure 6c). We next quantified cell death of vehicle control- or drug combo-treated HCT-116 K-RAS (WT/G13D) cells by the flow cytometric propidium iodide (PI) exclusion assay. We found an increase in PI-positive (PI+ve) HCT-116 cells, which is likely due to the loss of cell membrane integrity when these cells were exposed to the drug combo (Figure 6d). Remarkably, there was a marked reduction in PI+ve cells when these cells were co-incubated with the drug combo and the pan-caspase inhibitor, Z-VAD-FMK (Figure 6d). Consistent with activation of apoptosis, cleavage of PARP and caspase 3 was observed in the drug combination-treated RAS-mutant cancer cells (Figure 6e).

CK1α and the proteasome may regulate the turnover of a myriad of proteins. To test whether FOXO4 is a key mediator of RAS-mutant cancer cell growth arrest and death, we transfected small amounts of the FLAG-tagged wildtype FOXO4 (FLAG-FOXO4^WT^) or CK1α-resistant mutant of FOXO4 (FLAG-FOXO4^S265/268A^) in RAS-mutant HCT-116 and SW480 colon cancer cells to test if they are sufficient to arrest cell growth. We showed that, while the ectopic expression of FLAG-FOXO4^WT^ led to a modest reduction of RAS-mutant colon cancer cell growth even at the highest plasmid amount, an equal amount of FLAG-FOXO4^S265/268A^ was ~30-fold more effective (30 ng of FOXO4^S265/268A^ plasmid was more effective than 1000 ng of wildtype plasmid; Figure 6f). Consistent with this, 30 ng of FLAG-FOXO4^S265/268A^ plasmid was sufficient to trigger PARP cleavage in these RAS-mutant colon cancer cells (Figure 6g). Taken together, our data strongly suggest that the D4476:Bortezomib-induced RAS-mutant cancer growth arrest/death is, in part, due to the blockade of FOXO4 tumour suppressor turnover.

## Discussion

Evasion of growth suppression is a key hallmark of cancer initiation and progression.^51^ Loss-of-function (LOF) somatic alterations or point mutations at tumour suppressor gene loci as well as aberrant transcriptional repression of their gene expression represent common mechanisms underlying evasion of growth suppression found in a multitude of human cancers.^52, 53, 54, 55, 56^ Although the FOXO family of longevity-promoting transcription factors has been shown to be bona fide tumour suppressors by an increasing number of reports, LOF somatic alterations or point mutations at *FOXO* gene loci and transcriptional silencing of *FOXO* gene expression rarely occur in human cancers, regardless of whether aberrant activation of RAS or its downstream effectors exists (Supplementary Figure 1a–d). Given that the activation of RAS or its downstream effectors induces post-translational modification of FOXO proteins to alter their subcellular localization and cellular function, we hypothesize that targeting FOXOs at the protein level is an exclusive route to overcome growth suppression in RAS-mutant cancer cells.

**Figure 7:**
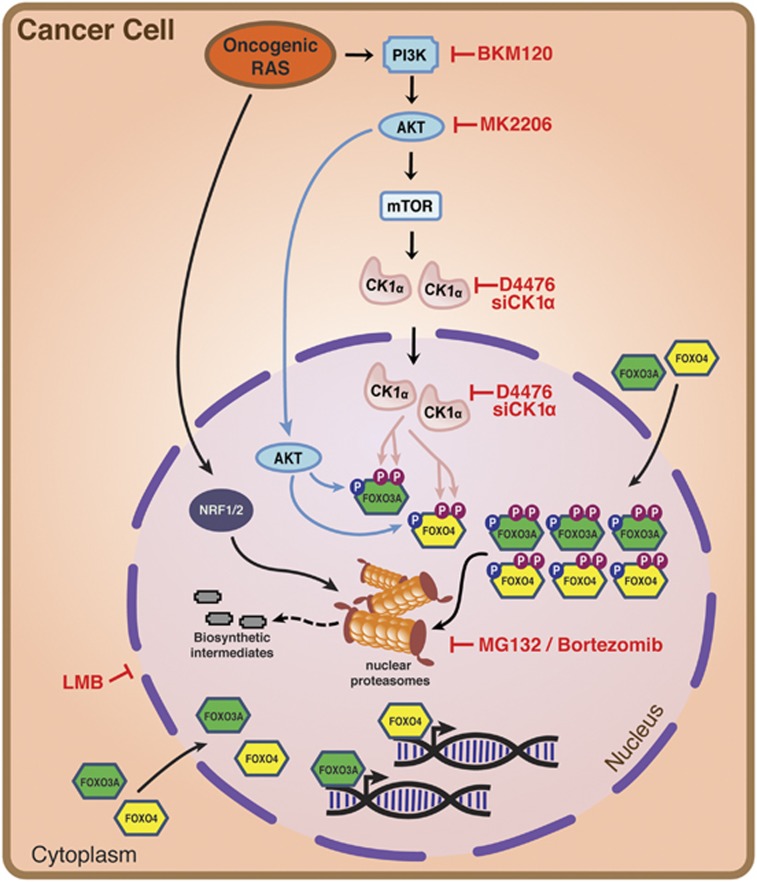
A model of CK1α-, 26 S proteasome-dependent turnover of FOXO tumour suppressors in RAS-mutant cancer cells. Oncogenic RAS, via its downstream PI3K/AKT/mTOR effector pathway, increases CK1α protein abundance. Sequential phosphorylation of specific serine residues of FOXO3A and FOXO4 tumour suppressors by AKT and CK1α earmark them for further modifications, leading to their proteolytic degradation by nuclear proteasomes. Oncogenic RAS also drives Nrf1/2-dependent expression of proteasome subunits to elevate abundance and activity of the 26 S proteasomes for proteolytic eradication of nuclear FOXO3A and FOXO4 from RAS-mutant cancer cells. Blockade of CK1α abundance or activity (by RNAi or D4476), or inhibition of 26 S proteasome stabilizes FOXO4 to induce caspase-dependent apoptosis of RAS-mutant cancer cells.

We have previously shown that mutant RAS, via its PI3K/AKT/mTOR effector signalling axis, upregulates the protein abundance of CK1α, leading to phosphorylation-driven destabilization of nuclear FOXO3A in RAS-mutant cancer cells.^29^ In the present study, we further showed that CK1α proteins are upregulated in both cytoplasm and nuclei of RAS-mutant cancer cells (Supplementary Figure 7a). Importantly, we demonstrate that CK1α phosphorylates and primes the less well-characterized FOXO4 tumour suppressor for proteolytic degradation, suggesting that RAS-mutant cancer cells have evolved to use CK1α phosphorylation-driven proteolysis as a general mechanism to specifically eradicate the FOXO family of tumour suppressors in their nuclei (Figure 7). This is consistent with the known roles of CK1α in regulating transcription factors and other substrates that function primarily in the nucleus.^32, 57, 58, 59^ Corroborating the *in silico* phosphorylation motif conservation and function prediction, we showed that the CK1α-mediated phosphorylation of FOXO4 at its Ser-265/268 residues is dependent on the priming phosphorylation at its Ser-262 residue by the PI3K/AKT signalling axis in HEK293 and HCT-116 cells. Although our data is consistent with published literature that reported AKT-mediated phosphorylation of FOXO4 in NIH3T3 cells,^25^ the mechanism to disrupt nuclear FOXO4 function appears to differ in a cell/tissue-specific manner. It is well documented that AKT-mediated phosphorylation of FOXO proteins induces the recruitment of 14-3-3 to FOXOs, leading to CRM1-dependent nuclear exclusion and inhibition of FOXO transcriptional activities.^11, 12, 25, 60^ However, we found that endogenous FOXO4 proteins are predominantly localized to the nuclei of HCT-116 and SW480 cells at steady state, suggesting that cytoplasmic sequestration of FOXO4 by 14-3-3 might be impaired in these cells. Since forced expression of 14-3-3 has been shown to stabilize FoxO3 proteins by blocking their degradation,^61^ we assessed whether 14-3-3 proteins are differentially expressed in the HCT-116 K-RAS isogenic cell lines. We showed that 14-3-3 proteins remain highly abundant in these cells, although they appeared to be marginally reduced in HCT-116 cells with the mutant K-RAS allele (Supplementary Figure 7b). Our finding argues that 14-3-3-mediated nucleo-cytoplasmic shuttling of FOXO proteins might not play a dominant role in regulating the functions of FOXOs in HCT-116 cells, and possibly other RAS-mutant cancer cells. Alternatively, the reduction in 14-3-3 protein abundance as a result of mutant RAS signalling might also lead to increased nuclear localization and CK1α-dependent destabilization of a subset of growth suppressors, such as the FOXOs.

Importantly, our data validated earlier studies involving RAS synthetic lethal screens that identified 26 S proteasome as the only common downstream survival-promoting effector in RAS-mutant cancer cells of diverse tissue origin.^45, 47, 50^ Our findings provide the first evidence to show that the presence of oncogenic RAS significantly enhanced nuclear proteasome activity. We found that expression of the *PSMA* family of core proteasome subunits is upregulated in a mutant RAS-specific manner. This might be due to increased expression of *Nrf1* and *Nrf2*, which are the key transcription factors that drive proteasome subunit expression and are known to be regulated by mutant RAS or its downstream effectors.^48, 49^ The mechanistic importance of this is supported by our observation that blockade of proteasome activity by MG132 or the FDA-approved Bortezomib is sufficient to increase FOXO3A and FOXO4 protein abundance in multiple RAS-mutant colon cancer cell lines.

Remarkably, while it is widely accepted that inhibition of CRM1-dependent nuclear export by LMB enhances nuclear p53 protein abundance,^62^ the abundance of nuclear FOXO3A and FOXO4 proteins was markedly reduced by LMB in HCT-116 and SW480 cells. Our data appear to be inconsistent with existing reports of nuclear retention and upregulation of FOXO isoforms in cancer cells by the clinical-grade nuclear export inhibitor, KPT-330 (also known as Selinexor).^63, 64, 65, 66, 67^ This discrepancy in the fate of nuclear FOXO3A/4 proteins could be attributed to the difference between persistent (LMB) versus transient (KPT-330) nuclear export inhibition.^68^ Since blockade of proteasome activity by Bortezomib reversed LMB-induced nuclear FOXO4 protein turnover, we speculate that nuclear proteasome degrades endogenous FOXO3A/4 proteins in RAS-mutant cancer cells. These data further substantiates the notion that CK1α-targeted tumour suppressors, such as p53 and FOXO3A/4, are regulated by different mechanisms in cancer cells. As a number of ubiquitin ligases target FOXOs,^69, 70, 71, 72^ our ongoing work also seek to determine which of these factors are required for the CK1α-, nuclear proteasome-dependent proteolysis of FOXO3A/4. We envisage that the CK1α-, nuclear proteasome-dependent proteolysis of FOXO3A/4 might be abolished in RAS-mutant cancer cells deficient in a FOXO3A/4-specific nuclear ubiquitin ligase. Alternatively, AKT- and/or CK1α-dependent phosphorylation of FOXO3A/4 might result in nuclear export and ubiquitination-mediated proteolysis by another yet unknown cytoplasmic ubiquitin ligase in these cancer cells.

Finally, the use of therapeutically effective but relatively high doses of FDA-approved proteasome inhibitors for cancer therapy can induce drug resistance,^73^ in which cancer cells trigger an adaptive response to overcome these inhibitors by enhancing Nrf1/2-driven proteasome gene transcription.^74, 75^ We suggest that this can be circumvented without reduction in therapeutic efficacy by combining lower dose Bortezomib with co-targeting of CK1α, an emerging key effector of RAS-mutant cancer cell survival. This is mimicked in part by the ectopic expression of FOXO4 (FOXO4^S265/268A^) that is resistant to CK1α-mediated proteolytic degradation, which also activates caspase-dependent apoptosis. It is likely that the synergy observed with the dual inhibition of CK1α and 26 S proteasome involves additional biological impairments in RAS-mutant cancer cells. In any case, this novel combination therapy may strike directly at the unique capability of RAS-mutant cancers to eradicate a specific subset of growth suppressors that are susceptible to kinase-driven proteolysis, such as the FOXO proteins. Consistent with the notion of attaining therapeutic efficacy by co-targeting of the PI3K/AKT/mTOR/CK1α signalling axis and 26 S proteasome, recent studies have shown that pharmacological inactivation of PI3K or mTOR enhances the sensitivity of cancer cells to Bortezomib and overcomes Bortezomib-induced resistance.^76, 77, 78^

In summary, our study identifies CK1α as a critical mediator of 26 S proteasome-dependent turnover of FOXO3A and FOXO4 tumour suppressors in RAS-mutant cancer cells. Inhibition of this kinase by the experimental drug D4476, combined with clinically approved proteasome inhibitors, accumulates nuclear FOXO4 to perturb the growth of RAS-mutant cancer cells by promoting apoptosis induction. The ability to target two essential nodes in RAS-mutant cancers via this paired therapy provides a new approach to treat this common subset of cancers.

## Materials and methods

### Plasmid DNA, cell culture and other reagents

The FLAG-FOXO4 expression construct was a gift from Domenico Accili (Addgene plasmid #17549). FLAG-FOXO4^S265A^, FLAG-FOXO4^S268A^, FLAG-FOXO4^S265/268A^ and siCK1α13-resistant HA-tagged, kinase-dead (K46A) CK1α expression constructs were created by the QuikChange^TM^ II XL Site-Directed Mutagenesis (SDM) Kit in accordance with the manufacturer’s instruction (Agilent Technologies, Inc., Santa Clara, CA, USA). Primer sequences used for SDM are described in Supplementary Table 1. The creation and use of pcDNA3.1-based siCK1α13-resistant HA-tagged, wildtype CK1α expression construct has been previously described.^29^ X-MAN isogenic HCT-116 colon cancer cell lines (mutant *K-RAS* wild-type/mutant *K-RAS*-null) were purchased from Horizon Discovery (Cambridge, UK) and cultured in accordance with the manufacturer’s instruction. All other human cancer cell lines used in this study were purchased from American Type Culture Collection (ATCC) and cultured in accordance with ATCC’s instruction. All cell lines were authenticated by vendors and tested negative for mycoplasma contamination in our lab before use. For cDNA overexpression in human cancer cell lines, X-tremeGENE HP DNA transfection reagent (Roche, Indianapolis, IN, USA) was used. Chemical compounds used in this study include BKM120 (S2247, Selleck Chemicals, Houston, TX, USA), Bortezomib (PS-341; S1013, Selleck Chemicals), Cycloheximide (CHX; C7698, Sigma Aldrich, St Louis, MO, USA), Dimethylsulfoxide (DMSO; D2650, Sigma Aldrich), D4476 (D1944, Sigma Aldrich), Leptomycin B (LMB; L2913, Sigma Aldrich), MK2206 (S1078, Selleck Chemicals), PF670462 (PF670; 3316, Tocris Bioscience), Propidium iodide (P4170, Sigma Aldrich), Z-Leu-Leu-Leu-al (MG132; C2211, Sigma Aldrich) and Z-VAD-FMK (V116, Sigma Aldrich).

### Reverse transcription-qPCR analysis

Total RNA was isolated from cultured cells using the RNeasy Mini Kit (74106, Qiagen, Hilden, Germany). Total RNA (2 μg) was reverse transcribed by the iScript Select cDNA Synthesis Kit (170–8896, Bio-Rad, Hercules, CA, USA) in accordance with the manufacturer’s instructions. Using 1/10 of the cDNA sample and independent qPCR primers targeting transcript of the indicated genes, qPCR was performed by the SsoFast EvaGreen Supermix Kit (172–5200, Bio-Rad) in accordance with the manufacturer’s instructions. Primer sequences used for RT-qPCR are described in Supplementary Table 1. Expression of the respective target transcript was quantified using qRT-PCR and normalized to β-actin and HPRT. Data are presented as fold-change relative to vehicle control. Data are mean±s.d. of experiments in triplicate and representative of three independent experiments.

### RNAi of human CK1α, CK1δ and CK1ε expression

Cells (2 × 10^5^) were plated in six-well plates and transfected with 100 nM non-targeting siRNA (siCtrl; D-001810-0X), human CK1α-, CK1δ-, or CK1ε-specific siRNAs (siCK1α, siCK1δ, or siCK1ε) using Dharmafect Transfection Reagent (Dharmacon RNAi Technologies, Lafayette, CO, USA), in accordance with the manufacturer’s instructions, for 48–72 h. The target sequences of human CK1α-, CK1δ- and CK1ε-specific ON-TARGETplus siRNAs (Dharmacon RNAi Technologies) have been previously reported.^29, 79^

### Denaturing SDS–polyacrylamide gel electrophoresis (SDS–PAGE) and western blot (WB) analysis

Cells were lysed by 4% SDS and total protein concentration was measured using the bicinchoninic acid protein assay kit (Thermo Scientific, Waltham, MA, USA). Proteins from whole cell extracts were resolved using denaturing SDS–PAGE and analyzed by WB. Primary antibodies used in WB analysis include anti-CK1α (UT3; in house), anti-CK1α (C-19; sc-6477, Santa Cruz Biotechnology Inc., Dallas, TX, USA), anti-CK1δ (128 A, Eli Lilly, Indianapolis, IN, USA), anti-CK1ε (610445, BD Biosciences, Franklin Lakes, NJ, USA), anti-FOXO1 (C29H4; #2880, Cell Signaling Technology (CST, Danvers, MA, USA), anti-FOXO3A (75D8; #2497, CST), anti-FOXO4 (ab128908, Abcam, Cambridge, MA, USA), anti-phospho-FOXO4^S262^ (ab126594, Abcam), anti-FLAG (M2) (F1804, Sigma Aldrich), anti-Lamin B (sc-6216, Santa Cruz Biotechnology Inc.), anti-GAPDH (#2118, CST), anti-Eg5 (4H3-1F12; #4203, CST), anti-β-tubulin (15568, Abcam), anti-p53 (DO-1; sc-126, Santa Cruz Biotechnology Inc.), anti-phospho-AKT^S473^ (#9271, CST), anti-AKT (#9272, CST), anti-poly ADP ribose polymerase (PARP; 556494, BD Biosciences) and anti-active caspase-3 (559565, BD Biosciences). The rabbit polyclonal phospho-FOXO4^S265/268^ antibody was synthesized at Abfrontier (Young In Frontier Co., Seoul, South Korea), using the phospho-peptide SNApSSVpSTRLSPLR-Cys. Secondary antibodies used in WB analysis include anti-mouse-/rabbit-/goat-horseradish peroxidase (Bio-Rad), anti-mouse-DyLight 800 and anti-rabbit-DyLight 680 (Thermo Fisher Scientific, Waltham, MA, USA). Images of immunoblot are acquired by the ImageQuant LAS 4000 system (GE Healthcare Life Sciences, Pittsburgh, PA, USA) or the LI-COR Odyssey Imaging system (LI-COR Biotechnology, Lincoln, NE, USA). Immunoblots shown in the accompanying figures are derived from three independent experiments. Loading controls used in the WB analysis include GAPDH, Eg5, β-tubulin, CK1α and AKT.

### Immunocytochemistry and immunoprecipitation

For immunocytochemistry assays, cells were grown to 50% confluence on glass coverslips in a 12-well tissue culture plate overnight. They were subsequently treated with the indicated chemical compounds at the indicated concentrations for the stated treatment duration. They were washed three times with 1 × PBS, fixed in pre-chilled 4% paraformaldehyde for 20 min, permeabilized in 0.1% Triton X-100 for 10 min and blocked with 3% bovine serum albumin (BSA) for 1 h. Primary immunostaining with the FOXO4 antibody or p53 antibody (DO-1) was performed at room temperature for 1 h, followed by immunostaining with Alexa Fluor 488- and Alexa Fluor 594-conjugated secondary antibody (Invitrogen, Thermo Fisher Scientific). Cellular DNA was subsequently counterstained with 40,6-diamidino-2-phenylindole (DAPI)-VectorShield (H-1200, Vector Laboratories, Inc., Burlingame, CA, USA). Staining was visualized and photographed using a LSM710 laser scanning confocal microscope with a × 63 oil immersion lens (Carl Zeiss Microimaging, Göttingen, Germany). Percent (%) nuclear FOXO4 staining in the drug-treated cells was scored by the nucleus counter plugin (automatic particle counting function) of FIJI Image J software in a double-blinded manner. Data are mean±s.d. of experiments in triplicate and representative of three independent experiments. For immunoprecipitation studies, cells were washed twice with ice-cold 1 × PBS (or 1 × TBS for experiments involving phosphoproteins), followed by cell lysis via gentle rocking in ice-cold modified radioimmune precipitation assay buffer (RIPA buffer: 50 mM Tris-HCl, pH 7.4, 1% Nonidet P-40, 0.25% sodium deoxycholate, 150 mM NaCl, 1 mM EDTA, 1 mM phenylmethylsulfonyl fluoride, 1 × cOmplete protease inhibitor cocktail (Roche) and 1 × PhosSTOP (Roche; for experiments involving phosphoproteins)). The cell lysates were pre-cleared with protein A/G plus-agarose beads (sc-2003, Santa Cruz Biotechnology Inc.) for 30 min prior to centrifugation at 14 000 *g* (4 °C, 15 min). The supernatants (500 μg per sample) were then incubated overnight with 1 μg FLAG (M2) antibody (F1804, Sigma Aldrich) at 4 °C with gentle tumbling. Each reaction mix was incubated with 20 μl protein A/G plus-agarose beads for 2 h at 4 °C with gentle tumbling and then precipitated via centrifugation at 1000 *g*, 4 °C, 5 min, followed by five washes with ice-cold modified RIPA buffer. The beads were then resuspended in 60 μl of 2 × WB sample loading buffer and boiled for 5 min prior to denaturing SDS–PAGE and WB analysis.

### Subcellular fractionation

Subcellular fractionation of cells was performed in the presence of 1 × cOmplete protease inhibitor cocktail using the NE-PER Nuclear and Cytoplasmic Extraction Kit (PI-78835, Thermo Scientific) in accordance with the manufacturer’s recommendation.

### *In vitro* proteasome activity assay

Cells were scrapped from culture flasks and washed twice with ice-cold 1 × PBS, prior to centrifugation at 300 *g*, 4 °C, 5 min, to isolate the cell pellet. The cell pellet was lysed in 0.5 ml of lysis buffer, which contains 50 mM HEPES (pH 7.5), 5 mM EDTA, 150 mM NaCl, 1 mM DTT, 1% Triton X-100 and 1 × cOmplete protease inhibitor cocktail, for 30 min on ice, with brief vortex at 10 min intervals. Following the addition of 2 mM ATP to the lysate (2 mg/ml) to improve the recovery of intact 26 S proteasome, the lysate was centrifuged at 14 000 *g* (4 °C, 15 min) to isolate clear extract for *in vitro* proteasome activity assay. Forty microgram total protein per sample was assessed via the 20 S proteasome activity assay kit (APT280, Merck Millipore, Temecula, CA, USA) in accordance to the manufacturer’s recommendation. All nuclear and cytoplasmic fractions, which were used for *in vitro* proteasome activity assay, contained 1 mM DTT and 1 × cOmplete protease inhibitor cocktail.

### Crystal violet assay for determining viability of cultured cells

The use of crystal violet assay for determining viability of cultured cells has been previously described^80^ with the following modification. Crystal violet dye was released from the stained cells by incubating them with 1% SDS for 6 h at room temperature with gentle shaking, followed by optical density (OD) 595 nm measurements of the released dye via the Benchmark Plus microplate absorbance reader (Bio-Rad). The two-color heat maps, which represent the percentage (%) of cancer cell growth inhibition of the drug treated groups (relative to the vehicle control-treated group), were generated by the Conditional Formatting function of Microsoft Excel software. Percent (%) cancer cell growth inhibition of the drug treated groups (relative to the vehicle control-treated group) is also represented by scatter plots using the GraphPad Prism software (La Jolla, CA, USA).

### Flow cytometry cell death analysis

Analysis of apoptosis by propidium iodide (PI) staining and flow cytometry has been previously described.^81^ Three independent experiments (with triplicate) were performed, in which 30 000 cells from each treatment group were analyzed via flow cytometry for PI uptake as a result of loss of membrane integrity in apoptotic cells.

### Statistical analysis

Statistical significance in experiments was assessed using GraphPad Prism, version 5 (GraphPad Software). Student’s Unpaired, two-tailed *t*-tests with a 95% confidence interval (CI) were used to analyze the data involving direct comparison of an experimental group with a control group. One-way ANOVA tests with Dunnett’s method for multiple comparisons with a 95% CI were used to analyze the data involving 2 or more test groups and a control group. One-way ANOVA tests with Bonferroni’s method with a 95% CI were used to analyze the data involving multiple pairwise comparisons of test and control groups. A *P*-value <0.05 was considered statistically significantly. All experiments were performed with biological (*n*=3) and technical (*n*=3) replicates, unless stated.
