# Supplementary material for: Oncogenic RAS-induced CK1α drives nuclear FOXO proteolysis
Source: Oncogene. 2017 Sep 25;37(3):363–76. doi: 10.1038/onc.2017.334 (PMC5799771; doi:10.1038/onc.2017.334)
Supplement: Supplementary Information [file onc2017334x1.docx]

**Oncogenic RAS-induced CK1α drives nuclear FOXO proteolysis**

Fuquan Zhang^1^, David M Virshup^1,2,3,4^ and Jit Kong Cheong^1,4^

^1^Programme in Cancer and Stem Cell Biology, Duke-NUS Medical School, Singapore. ^2^Department of Biochemistry, National University of Singapore, Singapore. ^3^Department of Pediatrics, Duke University School of Medicine, Durham, NC, USA. ^4^Address correspondence to: David M Virshup or Jit Kong Cheong, Programme in Cancer and Stem Cell Biology, Duke-NUS Medical School, Singapore. 8 College Road, 169857, Singapore. Phone: +65-65166954; Fax: +65-62212402; Email: [david.virshup@duke-nus.edu.sg](mailto:david.virshup@duke-nus.edu.sg) or [jitkong.cheong@duke-nus.edu.sg](mailto:Jitkong.cheong@duke-nus.edu.sg)

**Supplemental information**

**Materials and Methods**

*Copy number alterations, mutations and expression analysis of human cancer genomes*

Human cancer genome datasets from The Cancer Genome Atlas (TCGA; colon, lung and pancreas) and the Broad Institute (multiple myeloma) were subjected to copy number alterations, mutations and expression (RNA-Seq) analysis via the cBioPortal for Cancer Genomics (<http://www.cbioportal.org/>). The HUGO gene symbols *KRAS*, *HRAS*, *NRAS*, *BRAF*, *RALGDS*, *PIK3CA*, *MDM2*, *TP53*, *FOXO1*, *FOXO3*, *FOXO4*, *PTEN*, *APC*, *CDKN2A* and *CSNK1A1* were used for data query.

*In vitro alkaline phosphatase (ALP) assay*

*In vitro* ALP assay was performed in accordance to the manufacturer’s recommendation. 4 μg of protein from cell lysates were incubated with 10 Units of FastAP Thermosensitive Alkaline Phosphatase (EF0654, Thermo Scientific) at 37^o^C for 1 h, followed by addition of 50 mM EDTA to the reaction mix to terminate the reaction.

*In vitro kinase assays*

D4476 (single dose duplicate)-IC_50_ profiling of all human full-length recombinant CK1 isoforms was performed in a commercial assay (HotSpot Kinase, Reaction Biology Corp.). For *in vitro* GST-AKT kinase assays, HCT-116 cells were first transfected with EV, FLAG-FOXO4 WT or FLAG-FOXO4 (S262A) for 48 h. Cells were subsequently lysed by the cell lysis buffer [20 mM Tris (pH7.5), 150 mM NaCl, 1% Triton X-100, 1 mM EGTA, 1× cOmplete^TM^ protease inhibitor cocktail (Roche) and 1× PhosSTOP (Roche)]. FLAG-FOXO4 WT and S262A were immunoprecipitated by the FLAG (M2) antibody-bound Protein A/G plus-agarose beads. The immunoprecipitated FLAG-FOXO4 WT and S262A were then treated with 20 Units of FastAP Thermosensitive Alkaline Phosphatase (EF0654, Thermo Scientific) in 1× Alkaline Phosphatase buffer for 60 min at 37 °C, followed by three washes with the cell lysis buffer. The FastAP-treated FLAG-FOXO4 WT and S262A were subsequently incubated with 200 ng of recombinant human GST-AKT1 (A16-10G, SignalChem) in the kinase assay buffer [25 mM MOPS (pH 7.2), 25 mM MgC1_2_, 0.25 mM DTT and 1× PhosSTOP] for 30 min at 30 °C. MK2206 (1 μM) was pre-incubated with recombinant GST-AKT1 for 15 min at room temperature prior to addition to FastAP-treated FLAG-FOXO4 WT to serve as a control. For *in vitro* GST-CK1α kinase assays, the FastAP-treated FLAG-FOXO4 WT and S262A were first incubated with 200 ng of recombinant human GST-AKT1 in kinase assay buffer for 15 min at 30 °C, followed by three washes with the kinase assay buffer and incubation with 200 ng of recombinant human GST-CK1α1 (C64-10G, SignalChem) in kinase assay buffer for 30 min at 30 °C. D4476 (1 μM) was pre-incubated with recombinant GST-CK1α1 for 15 min at room temperature prior to addition to FastAP-treated, recombinant GST-AKT1-phosphorylated FLAG-FOXO4 WT to serve as a control.

**Supplemental figure legends**

**Supplemental figure 1. Somatic alterations and transcriptional dysregulation of CK1α and FOXOs are infrequent in RAS-mutant human cancers.** OncoPrints of (a) TCGA human colorectal adenocarcinoma (195 patients),^1^ (b) TCGA human NSC lung adenocarcinoma (230 patients),^2^ (c) TCGA human pancreatic adenocarcinoma (145 patients; Provisional) and (d) Broad human multiple myeloma (205 patients).^6^ Data query at cBioPortal for Cancer Genomics (http://www.cbioportal.org/) was performed using HUGO gene symbols *KRAS*, *HRAS*, *NRAS*, *BRAF*, *RALGDS*, *PIK3CA*, *MDM2*, *TP53*, *FOXO1*, *FOXO3*, *FOXO4*, *PTEN*, *APC*, *CDKN2A* and *CSNK1A1*. Data outputs include copy number alteration, mutation profile and fold change in gene expression (via RNA-Seq).

**Supplemental figure 2. CK1α regulates FOXO4 protein abundance in RAS-mutant cancer cells.** (a) D4476 is a CK1α/δ/ε-biased small molecule inhibitor. D4476-IC_50_ profiling of all human full-length recombinant CK1 isoforms was performed with the HotSpot Kinase miniaturized ^33^P radioisotope-based assay platform (Reaction Biology Corp.). D4476 was tested in 10-dose IC_50_ mode with a 3-fold serial dilution starting at 100 μM, in the presence of 10 μM ATP. % kinase activity was measured relative to the vehicle control (DMSO). D4476 dose-response curves were plotted, with the respective IC_50_ values determined, by the GraphPad Prism software. (b) RNAi depletion or pharmacological inhibition of CK1α increases FOXO4 protein abundance in SW480 colon cancer cells. For RNAi depletion of CK1α, cells transfected with 100 nM siCtrl or siCK1α (#13 or #14) for 48 h. To inhibit CK1α kinase activity, cells were treated with DMSO or D4476 (1 or 5 μM) for 16 h. Cells were then lysed for SDS-PAGE/WB. Protein blots from triplicated experiments were stained with the FOXO4, CK1α and GAPDH (loading control) antibodies. (c) Phosphorylation-associated gel mobility shifts of endogenous FOXO4 proteins in HCT-116 and SW480 cells were abolished by *in vitro* alkaline phosphatase (ALP) treatment. Protein blots from triplicated experiments were stained with the FOXO4 antibody. N.S: non-specific bands.

**Supplemental figure 3. The PI3K/AKT effector pathway of oncogenic RAS signaling regulates FOXO4 protein turnover.** (a) The PI3K-AKT signaling axis is required for FOXO4^S262^ phosphorylation. HEK293 cells were transfected with EV, WT or S262A for 48 h followed by IP with the FLAG (M2) antibody and SDS-PAGE/WB with the FOXO4 and p-FOXO4^S262^ antibodies. Note: The WT-transfected cells were treated with DMSO or 5 μM BKM120 (BKM) or MK2206 (MK) for 4 h prior to IP. WCL: Whole cell lysate. (b) Inhibition of PI3K or AKT abolished the phosphorylation-associated gel mobility shift of endogenous FOXO4 proteins. HCT-116 cells were treated with VC (vehicle control; DMSO or 10 μM BKM120 (BKM), MK2206 (MK) for 4 h prior to cell lysis and SDS-PAGE/WB. Protein blots from triplicated experiments in (a-b) were stained with the indicated antibodies. (c) Recombinant human GST-AKT1 phosphorylates FLAG-FOXO4 at S262. HCT-116 cells were transfected with EV, FLAG-FOXO4 WT or FLAG-FOXO4 (S262A) for 48 h. Cells were lysed by the cell lysis buffer, followed by immunoprecipitation of FLAG-FOXO4 WT and S262A via the FLAG (M2) antibody-bound Protein A/G plus-agarose beads. The immunoprecipitated FLAG-FOXO4 WT and S262A were pre-treated with Alkaline Phosphatase (FastAP; 20 Units) for 60 min at 37 °C, followed by incubation with Recombinant human GST-AKT1 (200 ng) for 30 min at 30 °C. MK2206 (1 μM) was pre-incubated with recombinant GST-AKT1 for 15 min at room temperature prior to addition to FastAP-treated FLAG-FOXO4 WT to serve as a control. Proteins in the reaction mix were subjected to SDS-PAGE/WB analysis with the indicated antibodies. (d) Recombinant human GST-CK1α1 phosphorylates FLAG-FOXO4 at S265 and S268. HCT-116 cells were transfected with EV, FLAG-FOXO4 WT or FLAG-FOXO4 (S262A) for 48 h. Cells were lysed by the cell lysis buffer, followed by immunoprecipitation of FLAG-FOXO4 WT and S262A via the FLAG (M2) antibody-bound Protein A/G plus-agarose beads. The immunoprecipitated FLAG-FOXO4 WT and S262A were pre-treated with FastAP (20 Units) for 60 min at 37 °C, followed by sequential incubation with recombinant human GST-AKT1 (200 ng; for 15 min at 30 °C) and recombinant human GST-CK1α1 (200 ng; for 30 min at 30 °C). D4476 (1 μM) was pre-incubated with recombinant GST-CK1α1 for 15 min at room temperature prior to addition to FastAP-treated, recombinant GST-AKT1-phosphorylated FLAG-FOXO4 WT to serve as a control. Proteins in the reaction mix were subjected to SDS-PAGE/WB analysis with the indicated antibodies. (e) CK1α-dependent FOXO4^S265/268^ phosphorylation requires AKT-mediated FOXO4^S262^ priming phosphorylation. HCT-116 cells were transfected with FLAG-FOXO4 WT for 48 h. Cells were lysed by the cell lysis buffer, followed by immunoprecipitation of FLAG-FOXO4 WT via the FLAG (M2) antibody-bound Protein A/G plus-agarose beads. The FLAG-FOXO4 WT beads was pre-treated with FastAP (20 Units) for 60 min at 37 °C, followed by incubation with recombinant human GST-CK1α1 (200 ng; for 15 min at 30 °C) or recombinant human GST-AKT1 (200 ng; for 15 min at 30 °C). For dual kinase treated samples, FLAG-FOXO4 WT beads was pre-incubated with GST-AKT1 in kinase assay buffer for 15 min at 30 °C, followed by addition of recombinant GST-CK1α1 (200 ng) and further incubation for 15 min at 30 °C. Proteins in the reaction mix were subjected to SDS-PAGE/WB analysis with the indicated antibodies. H/C: heavy chain of IgG. (f) Wildtype and phospho-acceptor mutants of FLAG-FOXO4 and HA-FOXO3A are predominantly localized to the nuclei of HCT-116 colon cancer cells. Representative FLAG (red):DAPI (blue) merged immunofluorescence images of HCT-116 cells, which were transfected with EV, FLAG-FOXO4 (WT, S262A, S265A, S268A or S265/268A) or HA-FOXO3A (WT, S315A, S318A, S321A or S318/321A) for 48 h, were shown. The indicated FLAG-FOXO4 WT-transfected HCT-116 cells were treated with LMB (20 nM), MK2206 (5 μM) or D4476 (5 μM), and the others were treated with DMSO vehicle control for 4 h prior to immunofluorescence staining using the FLAG (M2) antibody. DAPI stains the nuclei of cells. Scale bar: 10 μm. Similar results were observed in at least two independent experiments with replicates.

**Supplemental figure 4. Turnover of nuclear FOXO proteins is controlled by CK1α, the nuclear proteasome and the nuclear export machinery.** (a-b) Opposing effects of D4476, MG132 or Baf A and LMB on FOXO3A and FOXO4 protein abundance. (a) SW480 or (b) HCT-116 cells were treated with DMSO, D4476 (5 μM), MG132 (20 μM), Baf A (1 μM) or LMB (20 nM) for 4 h prior to cell lysis for SDS-PAGE/WB analysis. Protein blots from triplicated experiments were stained with the indicated antibodies (GAPDH and CK1α serve as the load controls). (c) Bortezomib upregulates FOXO3A protein abundance in a dose-dependent manner. HCT-116 cells were treated with vehicle control (Veh Ctrl; DMSO) or increasing doses of Bortezomib for 4 h prior to cell lysis for SDS-PAGE/WB analysis. Protein blots from triplicated experiments were stained with the FOXO3A and CK1α (loading control) antibodies. (d) Opposing effects of D4476, MG132 or Baf A and LMB on FOXO3A and FOXO4 protein abundance in the nuclei of HCT-116 cells. Cells were treated with DMSO, D4476 (5 μM), MG132 (20 μM), Baf A (1 μM) or LMB (20 nM) for 4 h prior to subcellular fractionation, followed by SDS-PAGE/WB analysis. Protein blots from triplicated experiments were stained with the indicated antibodies. Lamin B and GAPDH serve as the loading control for nuclear and cytosolic fractions respectively.

**Supplemental figure 5. LMB-induced degradation of nuclear FOXO proteins is reversed by CK1α or 26S proteasome inhibition.** (a) LMB-blockade of nuclear export reduced FOXO4 protein abundance in a time-dependent manner. SW480 cells were incubated with LMB (20 nM) over the indicated time, followed by cell lysis for SDS-PAGE/WB analysis. Protein blots from triplicated experiments were stained with the FOXO4, p53 (DO-1; positive control for LMB treatment) and GAPDH (loading control) antibodies. (b-c) LMB-blockade of nuclear export reduced FOXO3A/FOXO4 protein abundance in a time-dependent manner. (b) HCT-116 or SW480 cells were incubated with LMB (20 nM) over the indicated time, followed by cell lysis for SDS-PAGE/WB analysis. Protein blots from triplicated experiments were stained with the indicated antibodies. CK1α serves as the load control. (c) HCT-116 or SW480 cells were incubated with LMB (20 nM) over the indicated time prior to subcellular fractionation, followed by SDS-PAGE/WB analysis. Protein blots from triplicated experiments were stained with the indicated antibodies. Lamin B and β-tubulin serve as the loading control for nuclear and cytosolic fractions respectively. (d) HCT-116 or SW480 cells were treated with vehicle control (Veh Ctrl; ethanol) or LMB (20 nM), for 4 h prior to immunofluorescence staining using the FOXO4 and p53 antibodies. DAPI stains the nuclei of cells. Scale bar: 10 μm. p53-DAPI positivity (as a readout for nuclear p53) in each treatment group was quantified by automatic particle counting function of the FIJI Image J software. Data obtained was plotted using the GraphPad Prism software (mean ± SD). Student’s *t* test was used to analyze statistical significance; *****P* < 0.0001. (e) The CK1α-resistant FLAG-FOXO4^S265/268A^ is insensitive to proteasome-mediated proteolysis. HCT-116 cells were transfected with pBR322 empty vector (EV), pBR322-FLAG-FOXO4^WT^ (WT), or pBR322-FLAG-FOXO4^S265/268A^ (S265/268A) for 48 h, followed by treatment with 20 μM CHX or 20 μM CHX and 100 nM Bortezomib (Bort) for the indicated time. Cell lysates were used for SDS-PAGE/WB analysis. Protein blots from triplicated experiments were stained with the FLAG (M2) and GAPDH (loading control) antibodies. (f-g) Enhanced proteolytic degradation rate of endogenous FOXO3A is mutant RAS-specific. (f) HCT-116 K-RAS isogenic cell lines were incubated with DMSO or LMB (20 nM) over the indicated time followed by cell lysis for SDS-PAGE/WB analysis. Protein blots from triplicated experiments were stained with the FOXO3A and CK1α (loading control) antibodies. (g) Expression of FOXO3A is normalized with CK1α expression of each time point. Normalized FOXO3A expression in the LMB-treated samples of each cell line is plotted relative to that of its DMSO-treated samples using the GraphPad Prism software (mean ± SD). Similar results were observed in three independent experiments.

**Supplemental figure 6. Dual inhibition of CK1α and proteasome blocked RAS-mutant cancer cell growth.** (a) RAS-mutant HCT-116 cells possess higher nuclear proteasome activity. Subcellular fractionation of HCT-116 K-RAS isogenic cells was performed to isolate cytoplasmic and nuclear extracts (40 μg per extract) for fluorogenic substrate-based *in vitro* proteasome activity assays. Lactacystin (25 μM) treatment blocks proteasome activity. RLU: Relative light unit. One-way ANOVA with Bonferroni’s multiple comparisons test was used to analyze statistical significance; *****P* < 0.0001; not significant (n.s). (b) RAS-mutant cancer cells are selectively targeted by proteasome inhibition. HCT-116 K-RAS isogenic cells were treated with increasing doses of Bortezomib for 48 h, followed by crystal violet assays to assess cell viability. % cell growth of Bortezomib-treated samples of each cell line is plotted relative to that of its DMSO-treated samples using the GraphPad Prism software (mean ± SD). Similar results were observed in three independent experiments. (c) Dual CK1α and proteasome inhibition synergistically blocked the growth RAS-mutant cancer cells of diverse tissue origin. The indicated cell lines were treated with vehicle control (Veh Ctrl; DMSO), D4476 (1 or 5 μM) and/or Bortezomib (2 or 5 nM) for 72 h, followed by crystal violet assays to assess cell viability. % cancer cell growth inhibition of drug-treated groups were calculated relative to that of vehicle control-only (DMSO) group. One-way ANOVA with Dunnett’s test was used to analyze statistical significance; **P* < 0.05; ***P* < 0.01; *****P* < 0.0001; not significant (n.s).

**Supplemental figure 7. Comparison of protein abundance of CK1α and 14-3-3 in cytoplasmic and nuclear fractions of HCT-116 K-RAS isogenic cells** (a) CK1α is upregulated in both cytoplasmic and nuclear fraction of RAS-mutant HCT-116 cells. Subcellular fractionations of HCT-116 K-RAS isogenic cells were performed to isolate cytoplasmic and nuclear extracts for SDS-PAGE/WB analysis. Protein blots from triplicated experiments were stained with the CK1α, Lamin B (loading control for nuclear fraction) and GAPDH (loading control for cytoplasmic fraction) antibodies. (b) 14-3-3 proteins remain highly abundant in the cytoplasm of the HCT-116 K-RAS isogenic cells. Protein blots from triplicated experiments were stained with the pan-14-3-3, Lamin B (loading control for nuclear fraction) and β-tubulin (loading control for cytoplasmic fraction) antibodies. Similar results were observed in two independent experiments with triplicates.

**References**

1 Cancer Genome Atlas N. Comprehensive molecular characterization of human colon and rectal cancer. *Nature* 2012; **487:** 330-337.

2 Cancer Genome Atlas Research N. Comprehensive molecular profiling of lung adenocarcinoma. *Nature* 2014; **511:** 543-550.

3 Cheong JK, Nguyen TH, Wang H, Tan P, Voorhoeve PM, Lee SH *et al*. IC261 induces cell cycle arrest and apoptosis of human cancer cells via CK1delta/varepsilon and Wnt/beta-catenin independent inhibition of mitotic spindle formation. *Oncogene* 2011; **30:** 2558-2569.

4 Cheong JK, Zhang F, Chua PJ, Bay BH, Thorburn A, Virshup DM. Casein kinase 1alpha-dependent feedback loop controls autophagy in RAS-driven cancers. *J Clin Invest* 2015; **125:** 1401-1418.

5 Feoktistova M, Geserick P, Leverkus M. Crystal Violet Assay for Determining Viability of Cultured Cells. *Cold Spring Harb Protoc* 2016; **2016:** pdb prot087379.

6 Lohr JG, Stojanov P, Carter SL, Cruz-Gordillo P, Lawrence MS, Auclair D *et al*. Widespread genetic heterogeneity in multiple myeloma: implications for targeted therapy. *Cancer Cell* 2014; **25:** 91-101.

7 Riccardi C, Nicoletti I. Analysis of apoptosis by propidium iodide staining and flow cytometry. *Nat Protoc* 2006; **1:** 1458-1461.
