# Supplementary figures and images for: Oncogenic RAS-induced CK1α drives nuclear FOXO proteolysis
Source: Oncogene. 2017 Sep 25;37(3):363–76. doi: 10.1038/onc.2017.334 (PMC5799771; doi:10.1038/onc.2017.334)

# Suppl. Figure 1.

**a**

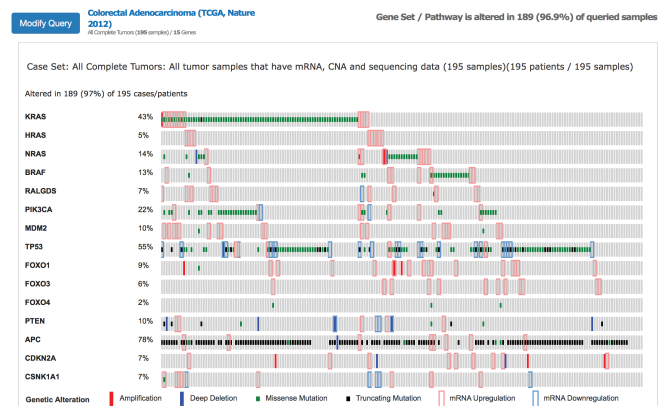

**b**

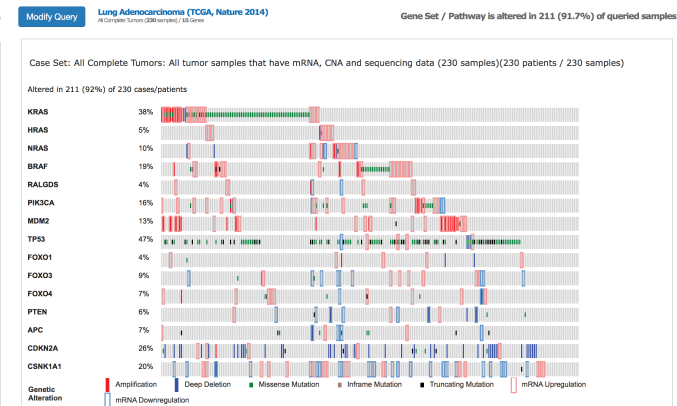

**c**

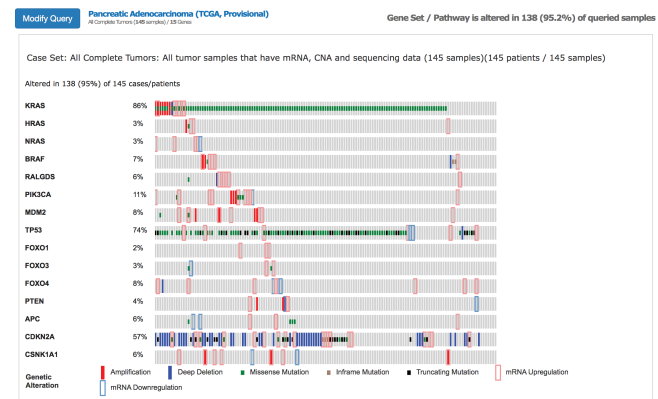

**d**

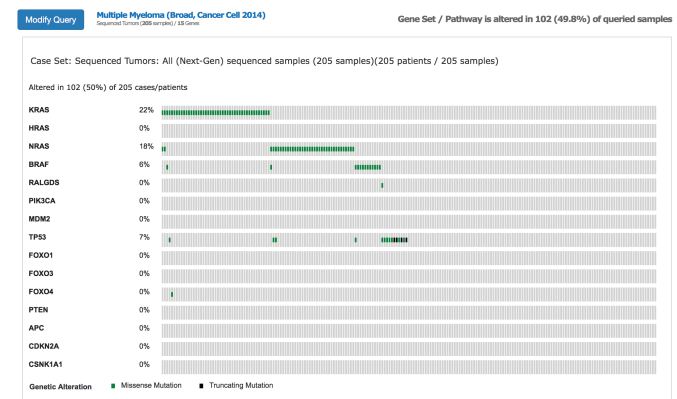

**a**

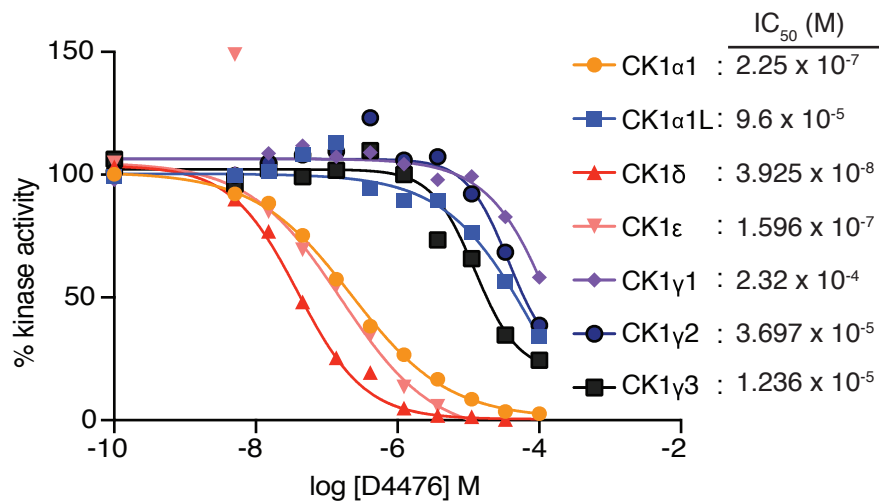

**b**

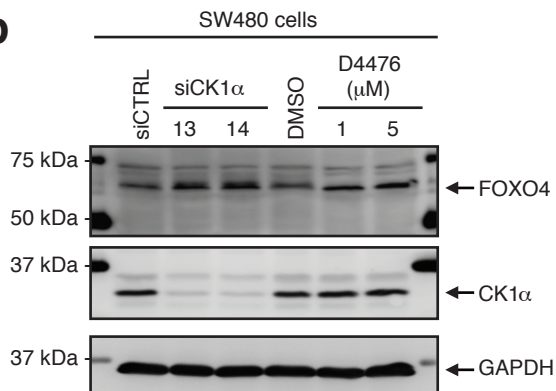

**c**

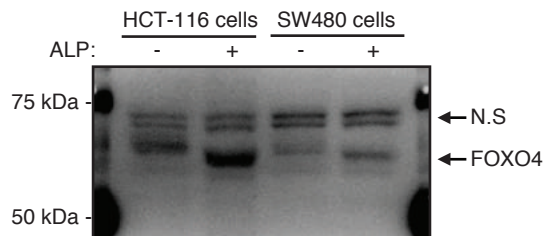

Suppl. Figure 3.

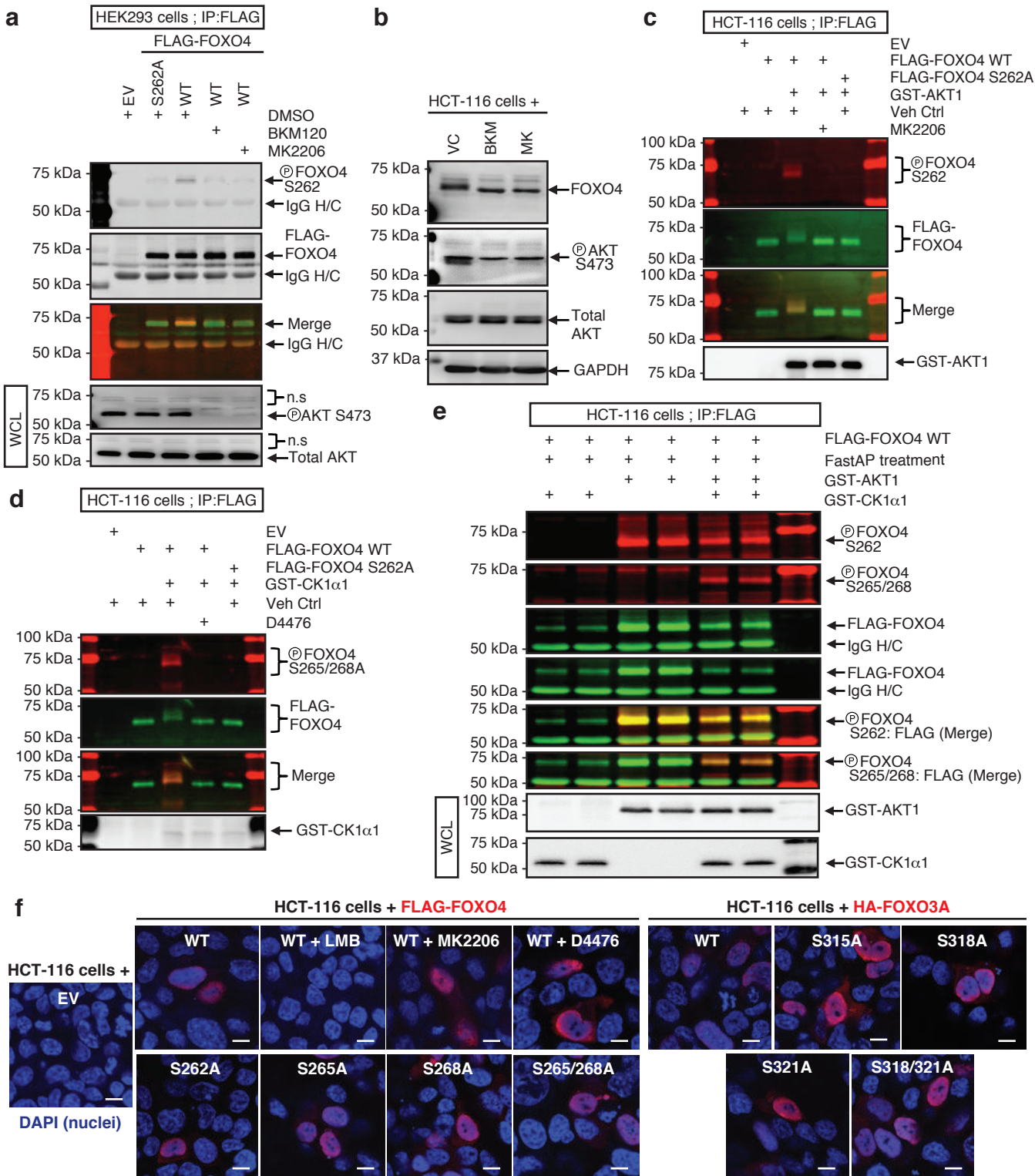

Suppl Figure 4.

**a**

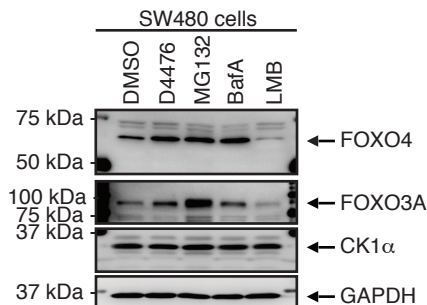

**b**

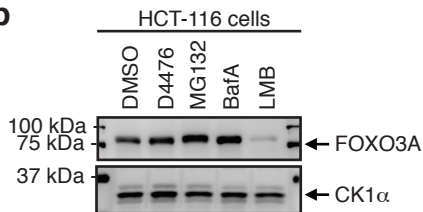

**c**

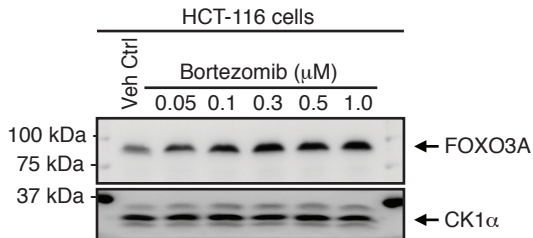

**d**

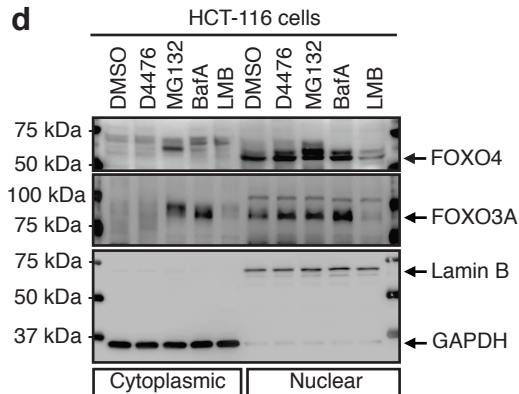

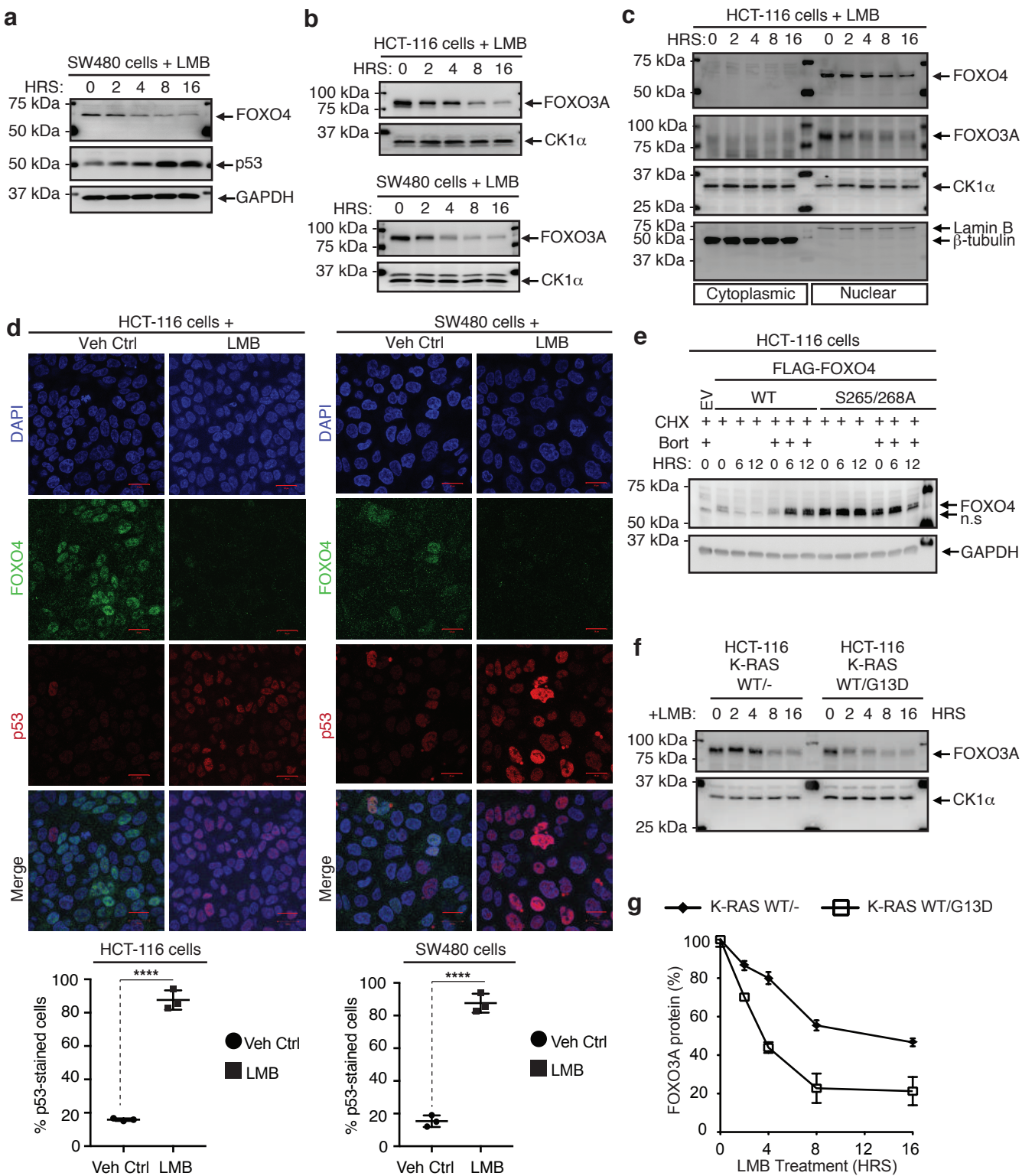

Suppl Figure 6.

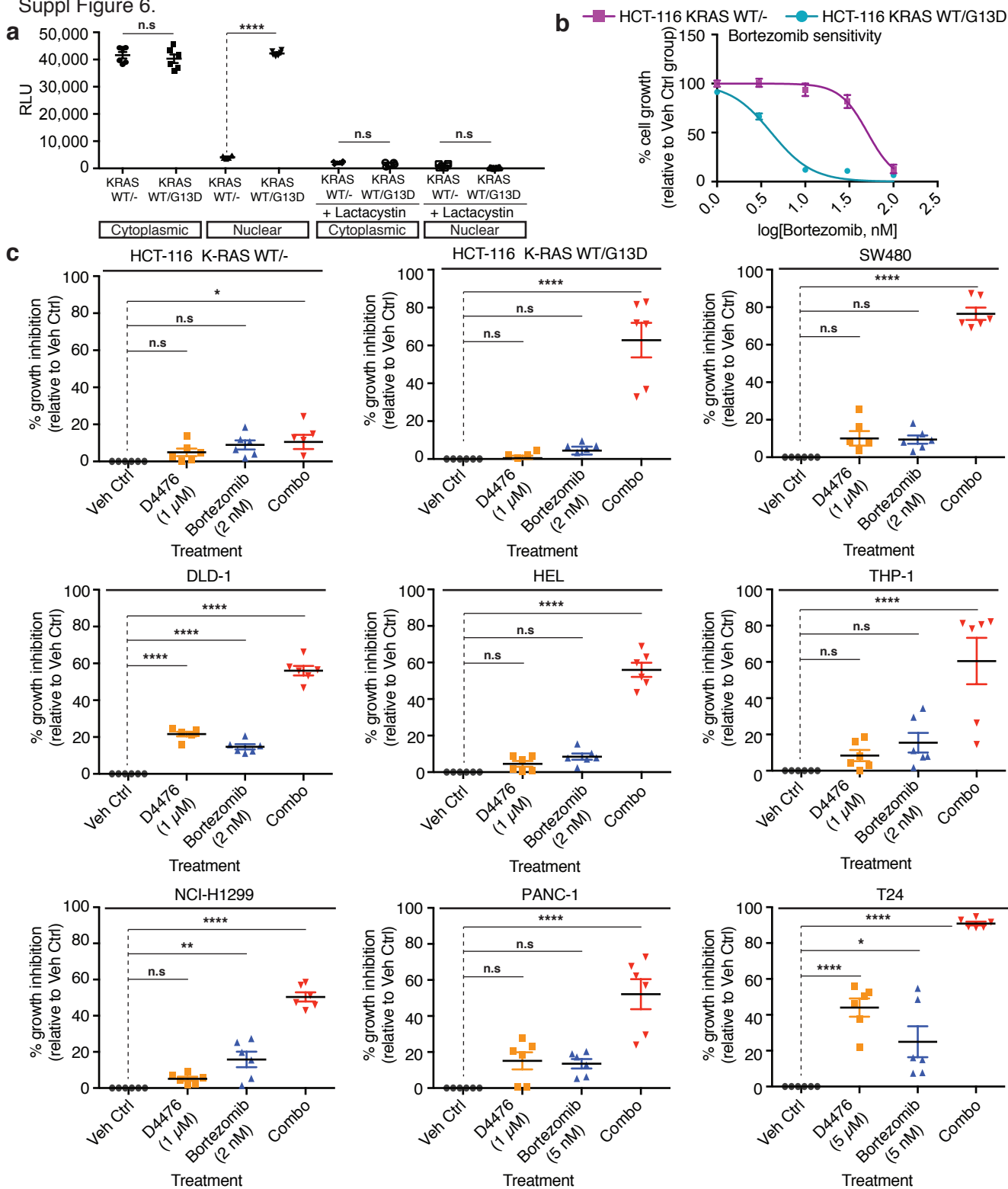

Suppl Figure 7.

**a**

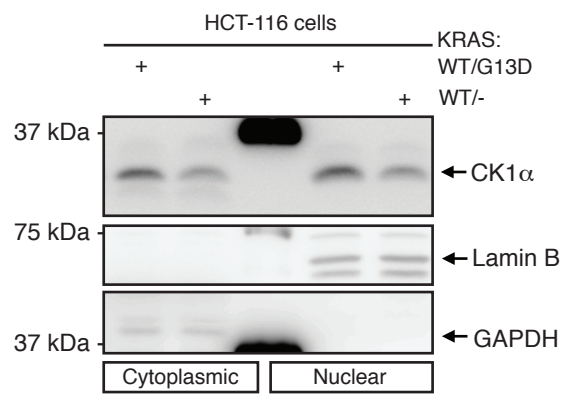

**b**

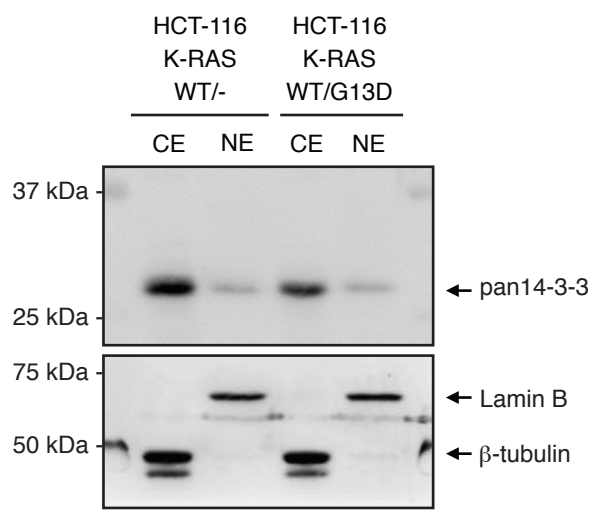

Supplement: Supplementary Figures [file onc2017334x2.pdf]
