# Supplementary material for: Oncogenic RAS-induced CK1α drives nuclear FOXO proteolysis
Source: Oncogene. 2017 Sep 25;37(3):363–76. doi: 10.1038/onc.2017.334 (PMC5799771; doi:10.1038/onc.2017.334)
Supplement: Supplementary Table 1 [file onc2017334x3.pdf]

Supplemental table 1. Sequence information of primers used in this study

Primers	Forward Sequence (5' to 3')	Reverse Sequence (5' to 3')
β-actin	AAGGATTCCTATGTGGGCGACG	GCCTGGATAGCAACGTACATGG
CK1α (K46A)	GCGAGGAAGTGGCAGTGGCGCTAGAATCTCAGAAGG	CCTTCTGAGATTCTAGCGCCACTGCCACTTCCTCGC
FOXO1	GCATCCATGGACAACAACAG	AGGCCATTTGGAAAACTGTG
FOXO3A	CTGAACTCCCTACGCCAGTC	GAAGTGAGCAGGTCCTGGAG
FOXO4	ACCGTGAAGAAGCCGATATG	CCTCAGACTCTGGCCTCAAG
FOXO4 (S262A)	CGTCCACGAAGCAGTGCAAATGCCAGCAGTGTC	GACACTGCTGGCATTTGCACTGCTTCGTGGACG
FOXO4 (S265A)	GCAGTTCAAATGCCGCCAGTGTCAGCACCCG	CGGGTGACACTGGCGGCATTTGAACTGC
FOXO4 (S268A)	CAAATGCCAGCAGTGTCGCCACCCGGCTGTCCCC	GGGGACAGCCGGGTGGCGACACTGCTGGCATTTG
FOXO4 (S265/S268A)	GCAGTTCAAATGCCGCCAGTGTCGCCACCCGGCTGTCCCC	GGGGACAGCCGGGTGGCGACACTGGCGGCATTTGAACTGC
HPRT1	CTCCGTTATGGCGACCC	CACCCTTTCCAAATCCTCAG
NRF1	AATCTGGAGCGTGATGTGGA	CCTTCTGCTTCATCTGTCGC
NRF2	CGGTATGCAACAGGACATTG	ACTGGTTGGGGTCTTCTGTG
PSMA1	AGACCAACTGTGGCTGAACC	GCAGCTCAGCAAGGTGAAGT
PSMA4	TCCATTCTCTCTTGTTAGTGTTGC	ATGACCTTGAAGTCAGCACTTG
PSMA5	GCCATTGAGGCTATCAAGCTTGC	AACTTCTTGCAAGGAGCTCTGGG
PSMA6	TGCACAGTCTTTCCCTCTGA	GTTTTGACCGCCACATTACC
PSMA7	TGGCCACTGACTTCTTCTCC	AAGTGGAGTACGCGCAGG
PSMB3	TGAGTTGAAGGAAGGTCGGC	TGGCTCAGTGTAGTAAGGGC
PSMB5	GAACGCATCTCTGTAGCAGC	CAGGGCCTCTCTTATCCCAG
PSMC1	TAACCGGCACTGAGTGTGAG	AAAGAAATATGAACCTCCTGTACCA
PSMC2	GGCAAACTGCTTTACGTTGA	GGATTTGGCTGCAGATAAGC
PSMC3	CCATCCAGCTGGTTCAGAAG	AGCGCCCTCTATCATCTTCA
PSMD12	GGCAGCCTCCATTTTACAGG	TCCTTCACAGCTAGGCAGAG
